# Polyanion Condensation in Inorganic and Hybrid Fluoridometallates (IV) of Octahedrally Coordinated Ti, Zr, Hf, V, Cr, W, Mn, Ge, Sn, and Pb

**DOI:** 10.3390/molecules29061361

**Published:** 2024-03-19

**Authors:** Zoran Mazej

**Affiliations:** Department of Inorganic Chemistry and Technology, Jožef Stefan Institute, Jamova Cesta 39, SI-1000 Ljubljana, Slovenia; zoran.mazej@ijs.si

**Keywords:** metal, fluoride, crystal structure, anion, octahedral coordination

## Abstract

In fluorides, the M^4+^ cations of M = Ti, V, Cr, Mn, Ge, Sn, and Pb favour the octahedral coordination of six F ligands. Some examples of M^4+^ with larger cations (M = Zr, Hf, W) in octahedral coordination are also known. If not enough F ligands are available to have isolated M^IV^F_6_ octahedra, they must share their F ligands. The crystal structures of such fluoride metalates (IV) show the variety of possible structural motifs of the zero-dimensional oligomeric anions [M_2_F_11_]^3−^ (M = Ti, Cr), [M_3_F_15_]^3−^ (M = Zr, Hf), [M_3_F_16_]^4−^ (M = Ge), [M_4_F_18_]^2−^ (M = Ti, W), [M_4_F_19_]^3−^ (M = Ti), [M_4_F_20_]^4−^ (M = Ti), [M_5_F_23_]^3−^ (M = Ti), [M_6_F_27_]^3−^ (M = Ti), [M_6_F_28_]^4−^ (M = Ti), [M_8_F_36_]^4−^ (M = Ti, Mn), [M_10_F_45_]^5−^ (M = Ti) to one-dimensional chains ([MF_5_]^−^)_∞_ (M = V, Ti, Cr, Ge, Sn, Pb), double chains ([M_2_F_9_]^−^)_∞_ (M = Ti, Mn), columns ([M_3_F_13_]^−^)_∞_ (M = Ti), ([M_4_F_19_]^3−^)_∞_ (M = Ti), ([M_7_F_30_]^2−^)_∞_ (M = Ti), ([M_9_F_38_]^2−^)_∞_) (M = Ti), two-dimensional layers ([M_2_F_9_]^−^)_∞_ (M = Cr), ([M_8_F_33_]^−^)_∞_ (M = Ti), and three-dimensional ([M_6_F_27_]^3−^)_∞_ (M = Ti) architectures. A discrete monomeric [M_2_F_9_]^−^ anion with two M^IV^F_6_ octahedra sharing a common face has not yet been experimentally demonstrated, while two examples containing discrete dimeric [M_2_F_10_]^2−^ anions (M = Ti) with two M^IV^F_6_ octahedra sharing an edge are still in question.

## 1. Introduction

In the structural chemistry of inorganic and hybrid (with organic cation and inorganic anion) fluorine compounds, the coordination number six with an octahedral coordination of the metal atom (M) of the anion by six fluorine ligands is preferred for almost all transition elements and for some main group elements [1,2]. Exceptions are metal cations of heavier elements, which prefer a higher coordination than six, and metal cations with the electron configurations d^8^ and d^9^, which often occur in square-planar coordination. In the fluorides, condensation of MF_6_ octahedra is favoured over the apexes, in contrast to the higher halogen homologues, where associations over the edges or faces are more common [3].

When the number of F ligands per M^IV^ cation is less than six, the M^IV^F_6_ octahedra must share their F ligands instead of being isolated. The usual term for such shared F atoms is a bridging fluorine atom (F_b_), i.e., the fluorine atom connects two metal centres of the anion. The term terminal fluorine atom (F_t_) is used for the remaining fluorine atoms that are not involved in such bridging.

The crystal structures of fluoride metallates (IV) with linked M^IV^F_6_ octahedra show the variety of possible structural motifs, from oligomeric anions to chains and columns to layers and three-dimensional framework architectures of the anions. This paper summarizes known perfluoridometallate (IV) salts with different anions determined in the crystal structures of inorganic and hybrid fluoridometallates (IV) with M = Ti, Zr, Hf, V, Cr, W, Mn, Ge, Sn, and Pb. Only examples with octahedral coordination of the M(IV) centre are included.

Many new inorganic and hybrid fluoridometallate (IV) salts of octahedrally coordinated Ti, Zr, Hf, V, Cr, W, Mn, Ge, Sn, and Pb have been structurally characterized in the last two decades. They contain anions in different sizes and geometries. Some of them were prepared for the first time and have a unique geometry. The aim of this review was to collect all of these data in one place and provide researchers with useful information for further planning of the preparation of new inorganic and hybrid fluoridometallate (IV) salts with anions in the desired geometry.

## 2. Discrete Oligomeric Anions

### 2.1. [M_2_F_9_]^−^ Anion (M = Ti, Ge)

**Figure 1:**
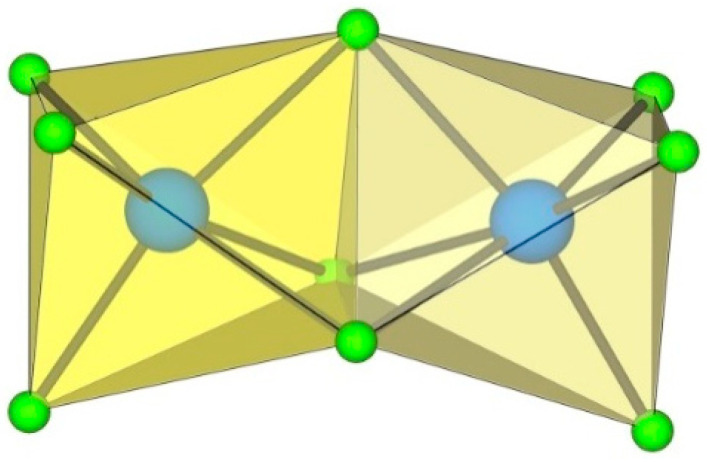
Hypothetical dimeric [M_2_F_9_]^−^ anion (M = Ti, Ge) with two M^IV^F_6_ octahedra sharing a common face.

^19^F NMR spectroscopy was used to detect the existence of the dimeric [Ti_2_F_9_]^−^ anion in liquid SO_2_ solution [4]. The [Ti_2_F_9_]^−^ anion has a face-linked bioctahedral structure (Figure 1). The “volume-based” thermodynamic approach suggests that cations larger than Cs^+^ favour the formation of solid perfluoridotitanium (IV) salts with discrete dimeric [Ti_2_F_9_]^−^ anions [5]. However, experiments have shown that an increase in the size of monocations does not favour the formation of [Ti_2_F_9_]^−^ over [Ti_4_F_18_]^2−^ salts (containing discrete anions). Crystal structure determination of the [Me_4_N]^+^ and [Ph_4_P]^+^ salts revealed that both compounds were [Ti_4_F_18_]^2−^ salts, i.e., [Me_4_N]_2_[Ti_4_F_18_] and [Ph_4_P]_2_[Ti_4_F_18_] were obtained instead of [Me_4_N][Ti_2_F_9_] and [Ph_4_P][Ti_2_F_9_] [6]. Although a theoretical ab initio study revealed that the dimeric [M_2_F_9_]^−^ anions (M = T, Ge) are predicted to be electronically and thermodynamically stable systems [7], all attempts to isolate salts with such anions in the solid state have failed so far.

### 2.2. [M_2_F_10_]^2−^ Anion (M = Ti)

**Figure 2:**
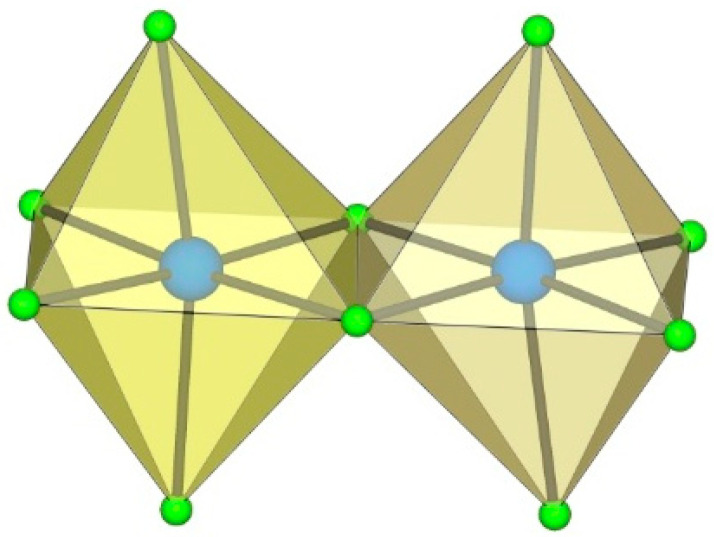
Hypothetical dimeric [M_2_F_10_]^2−^ anion (M = M^4+^) with two M^IV^F_6_ octahedra sharing a common edge.

The M^IV^F_6_ edge-sharing structure of the dimeric anion [Ti_2_F_10_]^2−^ was proposed on the basis of the ^19^F NMR data of the SO_2_ solution of the di-n-propylammonium hexafluoridotitanate–TiF_4_ system (Figure 2) [4]. Later, two crystal structures were described. However, both are doubtful. The first report describes a [Ti_2_F_10_]^2−^ salt of tetramethyltetrathiafulvalene (TMTTF), where the average charge of the single TMTTF cation was estimated to be +2/3, while the oxidation state of the titanium was assumed to be Ti^4+^ [8]. The second compound was originally formulated as a salt of the diprotonated piperazinium cation [C_4_H_12_N_2_]_2_[Ti_2_F_10_]·2H_2_O [9]. In this case, the anion has a charge of −4, which corresponds to a Ti^3+^ compound. Later, the formula was corrected by removing a hydrogen atom, resulting in a monoprotonated piperazinium cation [C_4_H_11_N_2_]_2_[Ti_2_F_10_]·2H_2_O [10]. In the figures shown, however, diprotonated cations remained [10]. The 3+ oxidation state is also indicated by the Jahn–Teller distortion of the octahedrally coordinated titanium atoms mentioned by the author. Therefore, the structure of the discrete dimeric [Ti_2_F_10_]^2−^ anion is still limited to the reported DFT-optimised theoretical structure [11], while reliable experimental evidence is still pending.

### 2.3. [M_2_F_11_]^3−^ Anion (M = Ti, Cr)

A summary of the crystal data of the salts consisting of [M_2_F_11_]^3−^ anions (M = Ti, Cr) is given in Table 1.

**Figure 3:**
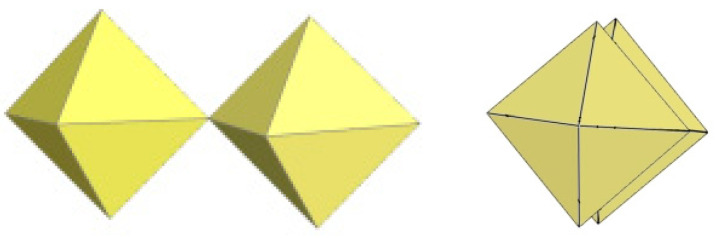

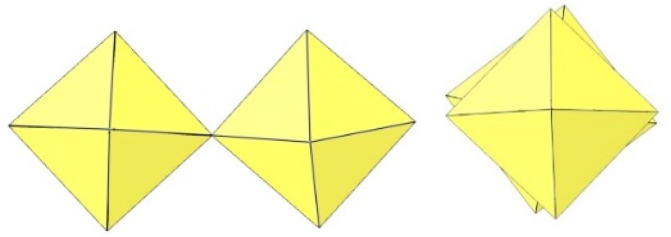
Two crystallographically different dimeric [Ti_2_F_11_]^−^ anions in the crystal structure of [C_3_H_5_N_2_]_3_[Ti_2_F_11_] with two TiF_6_ octahedra sharing a common vertex.

In discrete [M_2_F_11_]^3−^ anions (M = Ti, Cr), two MF_6_ octahedra share a common vertex. The distortion of the geometry of the [M_2_F_11_] units is usually described by the bridging angle α (bending of F_5_M–F_b_–MF_5_ around the bridging fluorine F_b_) and the torsion angle ψ (torsion of two planar MF_4,eq_ groups from the eclipsed to the staggered conformation). There are three crystallographically unique Ti_2_F_11_ units in [ImH]_3_[Ti_2_F_11_]. Each of them has a different conformation (Figure 3) [12]. In two of them, the equatorial TiF_4_-planes of the TiF_6_ octahedra of [Ti_2_F_11_]^3−^ are eclipsed and the Ti–F_b_–Ti angle is 180°. In the third, the TiF_4_-planes of two TiF_6_ octahedra are in gauche conformation with a dihedral angle of 8.50(6)° and a slightly bent Ti–F_b_–Ti angle (174.28(18)°) [12]. In both, [ImH]_3_[Ti_2_F_11_] and [C_5_H_6_N]_2_[H_3_O][Ti_2_F_11_]·H_2_O, the Ti–F_t_ bond lengths are comparable. They range from 1.768(3) to 1.908(2) Å for Ti–F_t_ bonds and from 1.9683(5) to 1.9805(6) Å for Ti–F_b_ bond lengths [12,13].

**Figure 4:**
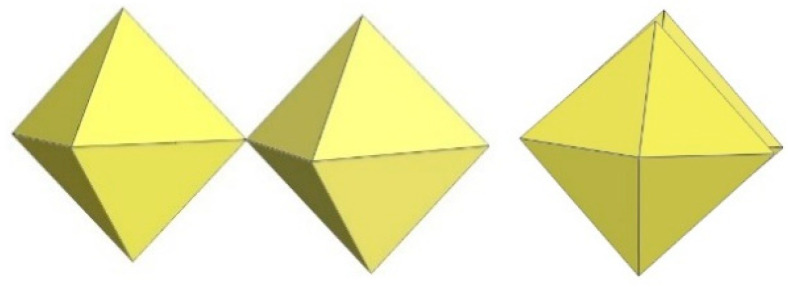
Dimeric [Ti_2_F_11_]^3−^ anion in the crystal structure of [C_5_H_6_N]_2_[H_3_O][Ti_2_F_11_]·H_2_O with two TiF_6_ octahedra sharing a common vertex.

In [C_5_H_6_]_2_[H_3_O][Ti_2_F_11_]·2H_2_O, the dimeric [Ti_2_F_11_]^3−^ anions are linear (Figure 4), nearly symmetric dimers (α = 180°; ψ is close to zero) [13].

The crystal structure of [N(CH_3_)_4_]_4_[Ti_2_F_11_][Ti_2_F_9_(H_2_O)_2_] contains disordered [Ti_2_F_11_]^3−^ anions [14]. The DFT optimized structure of the [Ti_2_F_11_]^3−^ anion has been also published [11]. The corner-sharing structure of the dimeric [Ti_2_F_11_]^3−^ anion was also proposed based on the ^19^F NMR data of the SO_2_ solution [4].

**Figure 5:**
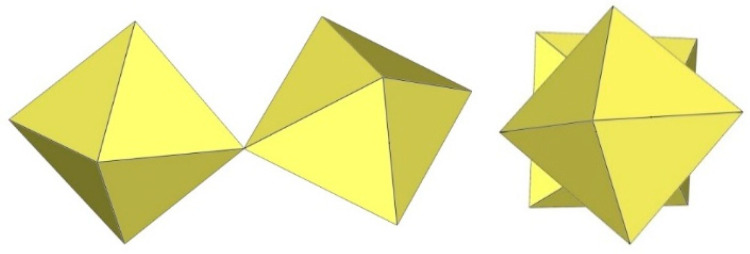
Dimeric [Cr_2_F_11_]^−^ anion in the crystal structure of K_3_Cr_2_F_11_·2HF with two CrF_6_ octahedra sharing a common vertex.

The crystal structure of K_3_Cr_2_F_11_·2HF shows [Cr_2_F_11_]^3−^ anions strongly distorted from the ideal D_4h_ symmetry (Figure 5) [15]. The bridging angle is 141° and the dihedral angle is 43° [15]. Due to the large Cr–F_b_–Cr bending angle, the fluorine atoms are in a staggered (gauche) conformation to minimize their repulsion. As expected, the Cr–F_t_ bonds are shorter (1.757 Å–1.817 Å) than the Cr–F_b_ bonds involved in the Cr–F_b_–Cr bridge (1.916(3) Å, 1.924(5) Å) or the Cr–F bonds involved in hydrogen bonding (1.901(5) Å) [15].

### 2.4. [M_3_F_13_]^−^ Anion (M = Ti, Ge)

**Figure 6:**
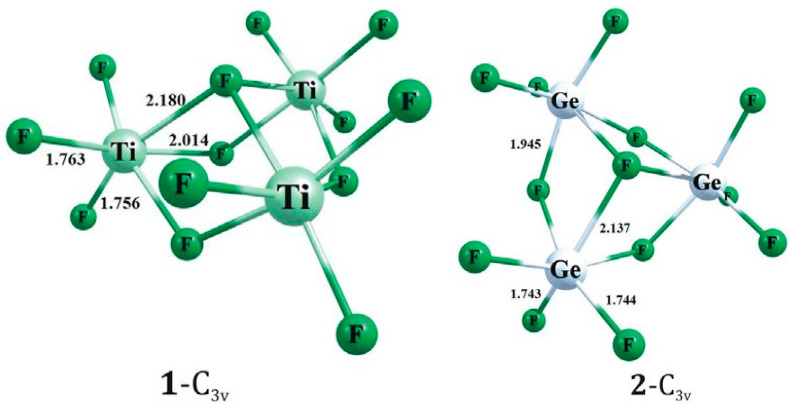
Theoretical models for trimeric [Ti_3_F_13_]^−^ anion (**left**) and [Ge_3_F_13_]^−^ anion (**right**). Copyright (2018) Elsevier. Used with permission from Ref. [7].

Theoretical ab initio calculations have shown that the global minimum structure of the [Ti_3_F_13_]^−^ anion corresponds to a C_3v_-symmetry structure comprising an equilateral triangle of three TiF_6_ octahedra that additionally share an F atom over the centre of the triangle (Figure 6) [7]. The entire structure can be considered as consisting of three octahedra sharing four F atoms. The oligomeric [Ti_3_F_13_]^−^ anion, the similar [Ge_3_F_13_]^−^ isomer, or another [M^IV^_3_F_13_]^−^ anion (M = M^4+^) have not yet been observed experimentally.

### 2.5. [M_3_F_15_]^3−^ Anion (M = Zr, Hf)

A summary of the crystal data of the salt consisting of [M_3_F_15_]^3−^ anions (M = Zr) is given in Table 2.

**Figure 7:**
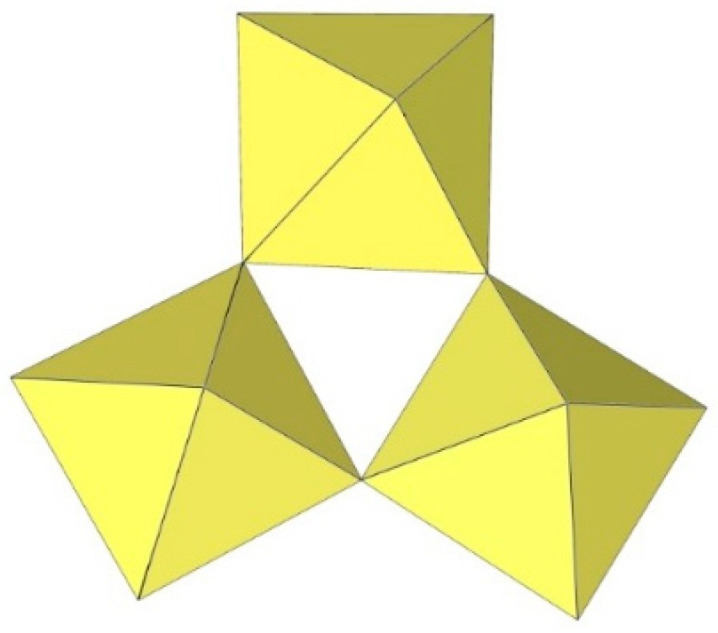
Trimeric [M_3_F_15_]^3−^ anion (M = Zr, Hf) in the crystal structure of [IDiPPH]_3_[M_3_F_15_]·4thf ·0.55(CH_2_Cl_2_) (IDiPP = 1,3-(2,6-di-isopropylphenyl)imidazol-2-ylidene).

The crystal structure of [IDiPPH]_3_[M_3_F_15_]·4thf·0.55(CH_2_Cl_2_) (M = Zr or Hf) (IDiPP = 1,3-(2,6-di-isopropylphenyl)imidazol-2-ylidene) consists of oligomeric trinuclear [M_3_F_15_]^3−^ (M = Zr, Hf) anions composed of three octahedral MF_6_ units sharing two cis-vertices and forming a triangle (Figure 7) [16]. In the Zr salt, the Zr–F_t_ bond lengths (1.942(4)–1.988(4) Å) are shorter than the Zr–F_b_ bond lengths (2.124(4)–2.139(4) Å), and the Zr–F_b_–Zr angles are in the range 155.6(2)–159.6(2)° [16]. The Hf system formed crystals of poor quality, so its complete crystal structure is not known.

### 2.6. [M_3_F_16_]^4−^ Anion (M = Ge)

A summary of the crystal data of the salts consisting of [M_3_F_16_]^4−^ anions (M = Ge) are given in Table 3.

**Figure 8:**
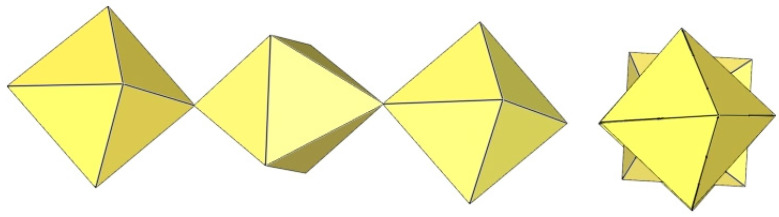
Trimeric [Ge_3_F_16_]^4−^ anion in the crystal structure of [(CH_2_)_2_SOH][Ge_3_F_16_].

**Figure 9:**
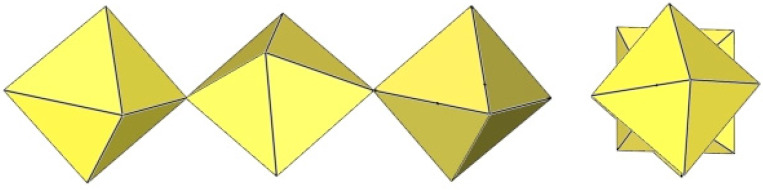
Trimeric [Ge_3_F_16_]^4−^ anion in the crystal structure of [C(NH_2_)_2_(NH_3_)_2_][Ge_3_F_16_]·2HF.

**Figure 10:**
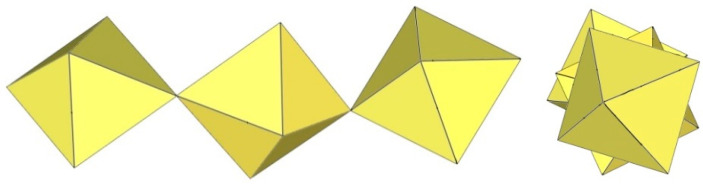
Trimeric [Ge_3_F_16_]^4−^ anion in the crystal structure of [C(NH_2_)_2_(NH_3_)_2_][Ge_3_F_16_]·HF.

Linear trimeric [Ge_3_F_16_]^4−^ anions were found in [(CH_2_)_2_SOH][Ge_3_F_16_] (Figure 8) [17], [C(NH_2_)_2_(NH_3_)_2_][Ge_3_F_16_]·HF (Figure 9) [18], and [C(NH_2_)_2_(NH_3_)_2_][Ge_3_F_16_]·2HF (Figure 10) [18]. The [Ge_3_F_16_]^4−^ anion consists of a chain of three slightly distorted GeF_6_ octahedra connected by the bridging F atoms in a staggered conformation. The F_b_ atoms are in the trans position. In [(CH_2_)_2_SOH][Ge_3_F_16_], as expected, the Ge–F_t_ bonds are shorter (1.744(3) Å–1.788(2) Å) than the Ge–F_b_ bonds (1.914(2)–1.921(2) Å) involved in the Ge–F_b_–Ge bridge [17]. The Ge–F_b_–Ge angles are in the range (144.8–149.9 Å) [17]. The corresponding bond lengths and angles in [C(NH_2_)_2_(NH_3_)_2_][Ge_3_F_16_]·nHF (*n* = 1, 2) are comparable [18].

### 2.7. [M_4_F_18_]^2−^ Anion (M = Ti, W)

A summary of the crystal data of the salts consisting of [M_4_F_18_]^2−^ anions (M = Ti, W) is given in Table 4.

**Figure 11:**
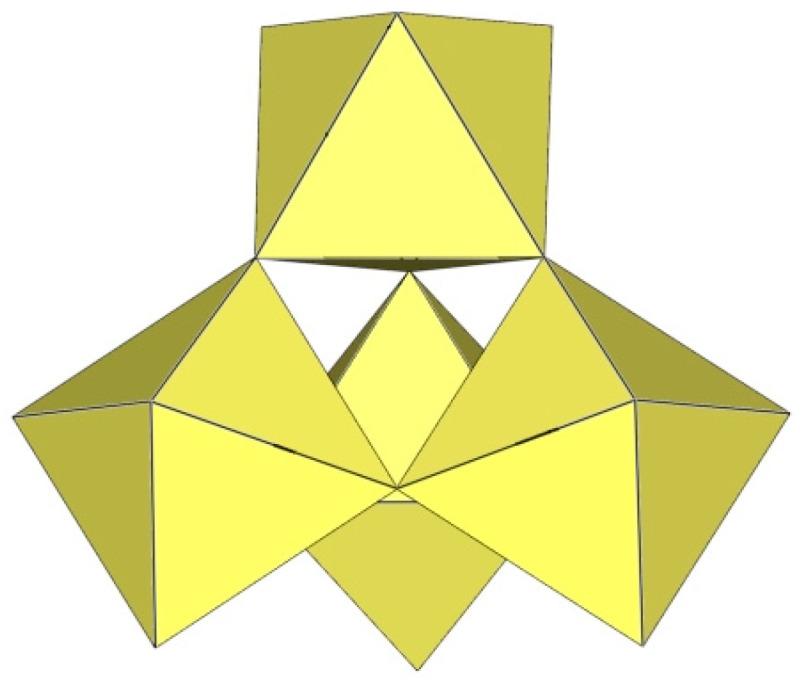
Tetrameric [M_4_F_18_]^2−^ anion (M = Ti, W) in the crystal structures of [TiF_2_([15]crown-5)][Ti_4_F_18_]⋅0.5MeCN, [N(CH_3_)_4_]_2_[Ti_4_F_18_], [(C_6_H_5_)_4_P]_2_[Ti_4_F_18_], [o-C_6_H_4_(P(C_6_H_5_)_2_H)_2_][Ti_4_F_18_], o-C_6_H_4_(As(CH_3_)_2_H)_2_][Ti_4_F_18_], [H^i^PrS(CH_2_)_2_S^i^PrH][Ti_4_F_18_], and [WCl_2_(cp)_2_][W_4_F_18_] (cp = η-C_6_H_5_).

The crystal structure of [TiF_2_([15]crown-5)][Ti_4_F_18_]⋅0.5MeCN was the first example of a tetrameric [Ti_4_F_18_]^2−^ anion (Figure 11) [5]. Later, it was also found in the salts [N(CH_3_)_4_]_2_[Ti_4_F_18_] [6], [(C_6_H_5_)_4_P]_2_[Ti_4_F_18_] [6], [o-C_6_H_4_(P(C_6_H_5_)_2_H)_2_][Ti_4_F_18_] [19], o-C_6_H_4_(As(CH_3_)_2_H)_2_][Ti_4_F_18_] [19], and [H^i^PrS(CH_2_)_2_S^i^PrH][Ti_4_F_18_] [19]. The latter two were only identified spectroscopically [19]. In the [Ti_4_F_18_]^2−^ anion, each TiF_6_ octahedron shares three of its F_b_ atoms (in the fac position) with three other TiF_6_ octahedra. Consequently, the Ti atoms of each TiF_6_ octahedron are coordinated by three terminal and three bridging fluorine atoms. The tetramer exhibits an overall T_d_ symmetry. The DFT-optimized structure of the [Ti_4_F_18_]^2−^ anion has also been reported [11].

The [W_4_F_18_]^2−^ anion in [WCl_2_(cp)_2_][W_4_F_18_] (cp = η-C_6_H_5_) has the same geometry as the [Ti_4_F_18_]^2−^ anion (Figure 11) [20]. The W–Ft bond distances range from 1.66(1) to 1.89(1) Å, while the W–F_b_ bond lengths are on average longer, ranging from 1.88(1) to 2.174(5) Å [20].

### 2.8. [M_4_F_19_]^3−^ Anion (M = Ti)

A summary of the crystal data of the salt consisting of [M_4_F_19_]^3−^ anions (M = Ti) is given in Table 5.

**Figure 12:**
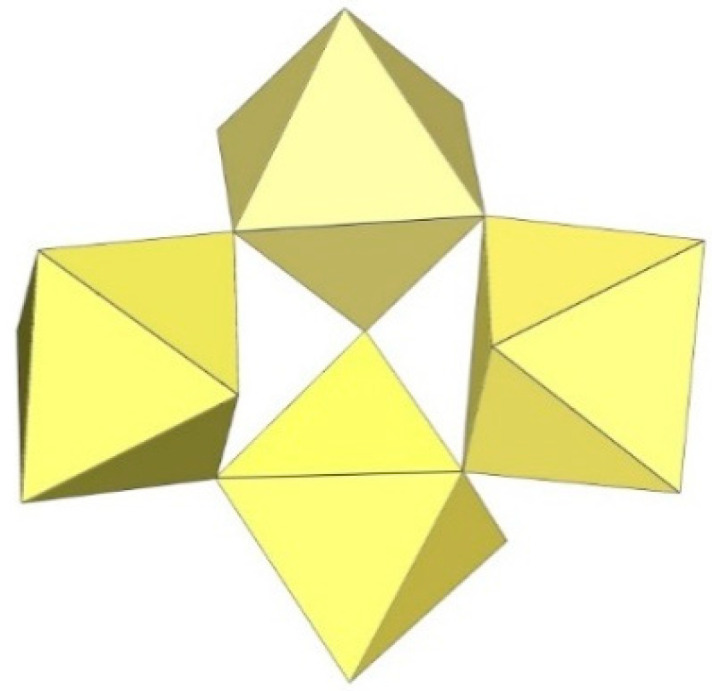
Tetrameric [Ti_4_F_19_]^3−^ anion in the crystal structure of [XeF_5_]_3_[Ti_4_F_19_].

The crystal structure of [XeF_5_]_3_[Ti_4_F_19_] is the only example containing discrete tetrameric [Ti_4_F_19_]^3−^ anions [21]. The [Ti_4_F_19_]^3−^ anion consists of four TiF_6_ octahedra. Two of the TiF_6_ octahedra, which share a fluorine atom, are additionally bridged by two TiF_6_ octahedra (Figure 12).

### 2.9. [M_4_F_20_]^4−^ Anion (M = Ti)

A summary of the crystal data of the salts consisting of [M_4_F_20_]^4−^ anions (M = Ti) is given in Table 6.

**Figure 13:**
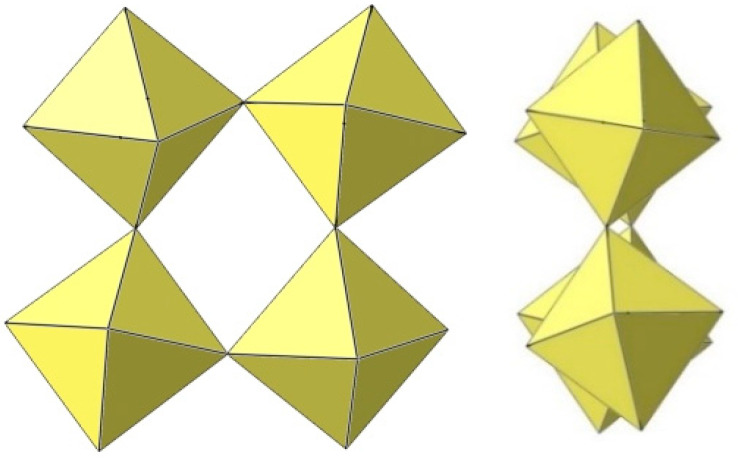
Tetrameric [Ti_4_F_20_]^4−^ anion in the crystal structure of α-[C_3_H_5_N_2_]_4_[Ti_4_F_20_].

**Figure 14:**
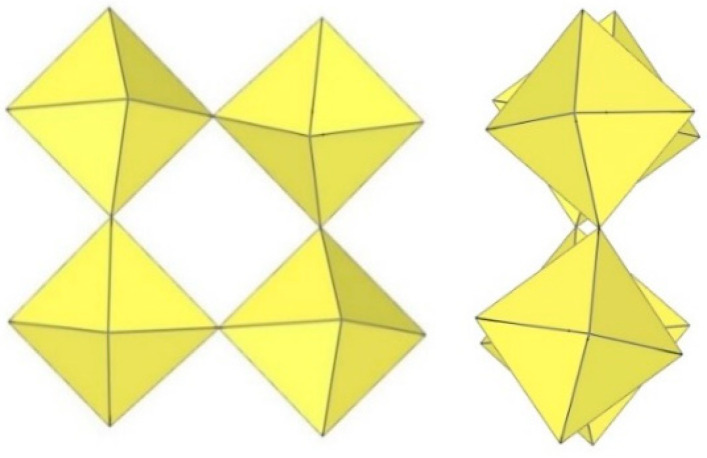
Tetrameric [Ti_4_F_20_]^4−^ anion in the crystal structure of β-[C_3_H_5_N_2_]_4_[Ti_4_F_20_].

**Figure 15:**
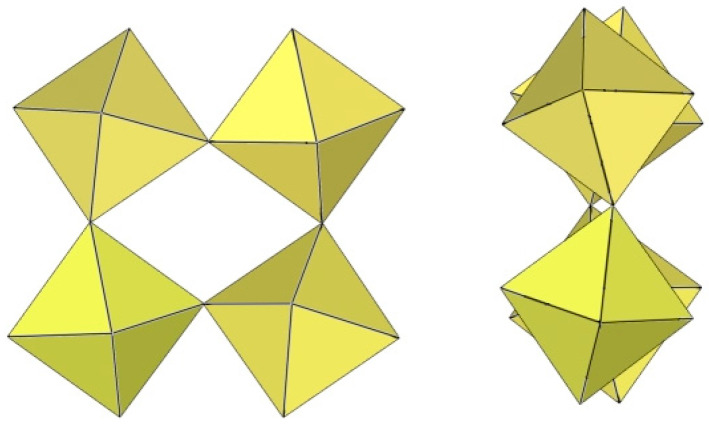
Tetrameric [Ti_4_F_20_]^4−^ anion in the crystal structure of [C(NH_2_)_3_]_4_[Ti_4_F_20_].

**Figure 16:**
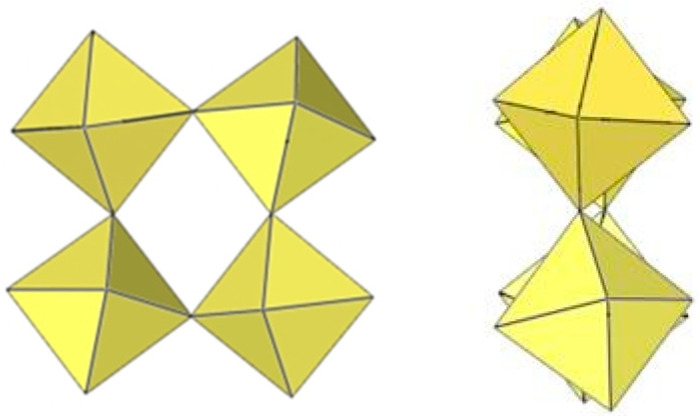
Tetrameric [Ti_4_F_20_]^4−^ anion in the crystal structure of [C(NH_2_)_3_]_4_(H_3_O)_4_[Ti_4_F_20_][TiF_5_]_4_.

The [Ti_4_F_20_]^4−^ anion consists of four TiF_6_ octahedra, which are connected to each other and form a slightly distorted planar square. Each octahedron shares two F atoms in the cis position. In all known examples (α- and β-[C_3_H_5_N_2_]_4_[Ti_4_F_20_] (Figure 13 and Figure 14), [C(NH_2_)_3_]_4_[Ti_4_F_20_] (Figure 15), and [C(NH_2_)_3_]_4_(H_3_O)_4_[Ti_4_F_20_][TiF_5_]_4_) (Figure 16), it has a similar geometry [12,22]. Each Ti atom is coordinated with two bridging and four terminal fluorine atoms. In β-[ImH]_4_[Ti_4_F_20_], the Ti–F_t_ bond lengths range from 1.776(3) to 1.824(4) Å and are significantly shorter than the Ti–F_b_ bonds (1.956(2) Å; 1.978(2) Å) [12]. The Ti–F_t_ and Ti–F_b_ bond lengths in other [M_4_F_20_]^4−^ salts [22] are comparable to those in β-[ImH]_4_[Ti_4_F_20_] [12]. Quantum chemical calculations at the B3LYP/SDDALL level of theory were used to determine the gas phase geometries and vibrational frequencies of the [Ti_4_F_20_]^4−^ anions, which was helpful in assigning the experimental vibrational frequencies of the anion [12].

### 2.10. [M_5_F_23_]^3−^ Anion (M = Ti)

A summary of the crystal data of the salt consisting of [M_5_F_23_]^3−^ anions (M = Ti) is given in Table 7.

**Figure 17:**
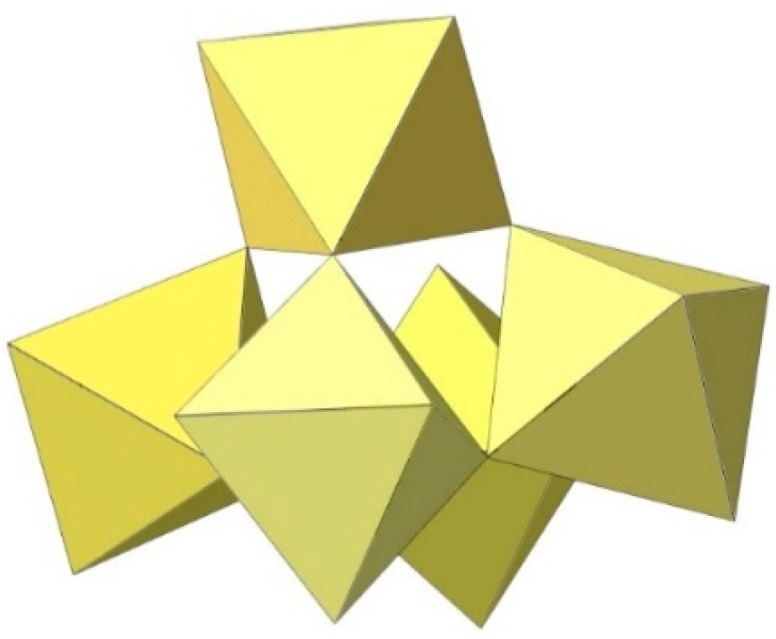
Pentameric [Ti_5_F_23_]^3−^ anion in the crystal structure of [ImH]_3_[Ti_5_F_23_].

The crystal structure of [ImH]_3_[Ti_5_F_23_] (Im = imidazole) is the only example that contains a discrete pentameric [M_5_F_23_]^3−^ anion (Figure 17) [12]. It is built from five TiF_6_ units, with four of the TiF_6_ octahedra sharing two cis-vertices and forming a tetrameric ring as in [Ti_4_F_20_]^4−^, and the fifth TiF_6_ unit sharing three fluorine vertices with three TiF_6_ units of the tetrameric ring. The bond lengths of Ti–F_t_ and Ti–F_b_ are 1.757(3)–1.848(3) Å and 1.942(2)–2.014(2) Å, respectively [12]. Quantum chemical calculations at the B3LYP/SDDALL level of theory were used to determine the gas phase geometries and vibrational frequencies of the [Ti_5_F_23_]^3−^ anions, which were helpful in assigning the experimental vibrational frequencies [12].

### 2.11. [M_6_F_27_]^3−^ Anion (M = Ti)

A summary of the crystal data of the salts consisting of [M_6_F_27_]^3−^ anions (M = Ti) is given in Table 8.

**Figure 18:**
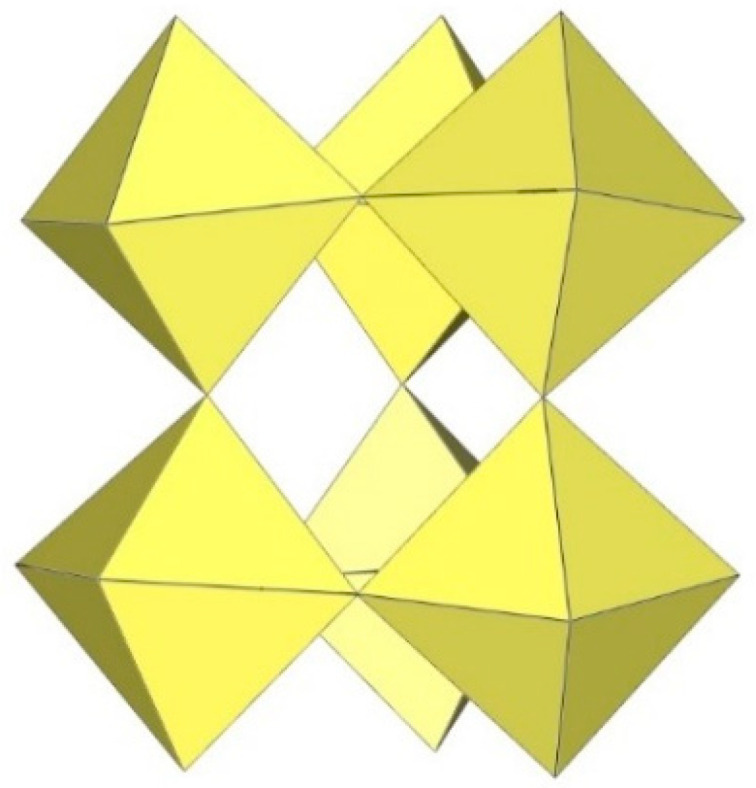
Hexameric [Ti_6_F_27_]^3−^ anion in the crystal structures of [C(NH_2_)_3_]_3_[Ti_6_F_27_]·SO_2_ and [C_3_H_5_N_2_]_2_[H_3_O][Ti_6_F_27_].

In C(NH_2_)_3_]_3_[Ti_6_F_27_]·SO_2_, the [Ti_6_F_27_]^3−^ anion consists of six TiF_6_ octahedra (Figure 18) [22]. Three TiF_6_ octahedra form a trimeric ring by sharing cis-vertices. Two such rings are connected via the bridging fluorine atoms and form a trigonal-prismatic geometry. In this way, all titanium atoms are coordinated with three F_t_ and three F_b_ atoms, which are located in the fac positions. The bond lengths of Ti–F_t_ and Ti–F_b_ are 1.754(1)–1.788(1) and 1.943(1)–2.010(1) Å, respectively [22].

The [Ti_6_F_27_]^3−^ anion with the same geometry was also observed in the crystal structure of [C_3_H_5_N_2_]_2_[H_3_O][Ti_6_F_27_] (Figure 18), where disordering of the imidazolium cations was observed and there were problems in determining additional cations providing the missing positive charge [22]. It was assumed that [H_3_O]^+^ cations were most likely present.

### 2.12. [M_6_F_28_]^4−^ Anion (M = Ti)

**Figure 19:**
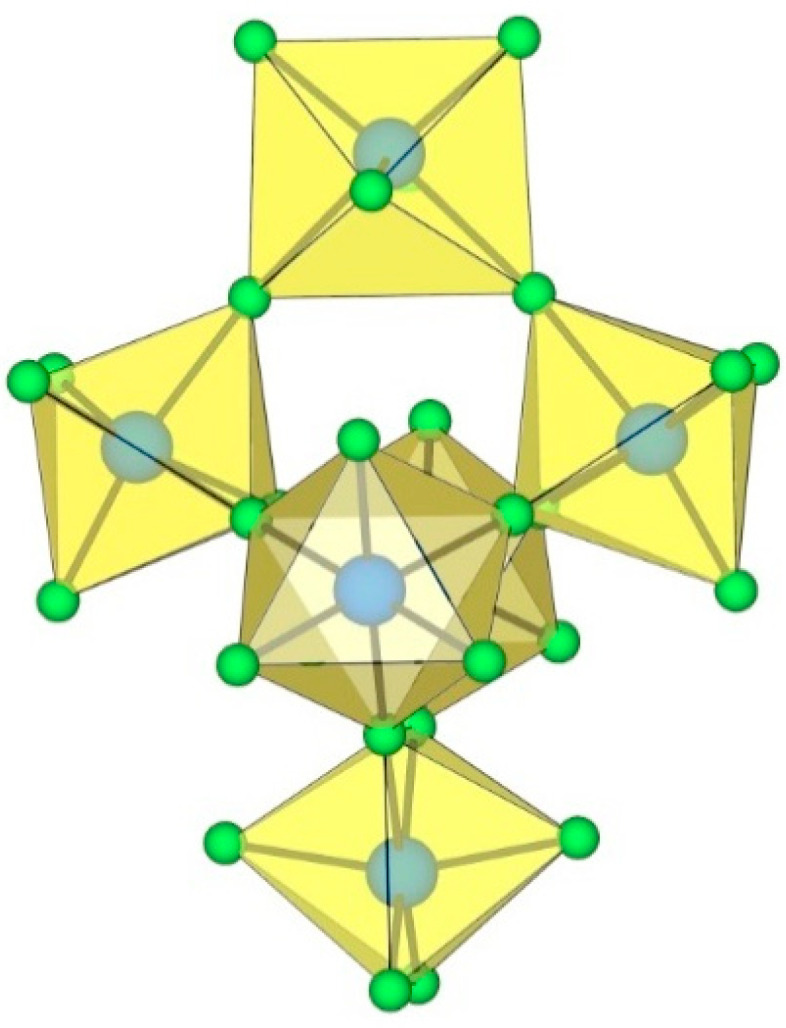
Hexameric [Ti_6_F_28_]^4−^ anion in the crystal structure of [ImH]_8−n_[X]_n_[Ti_8_F_36_][Ti_6_F_28_] (X = unknown cation).

In the study of the imidazole–TiF_4_-HF system, single crystals of the compound [ImH]_8−n_[X]_n_[Ti_8_F_36_][Ti_6_F_28_] were grown [23]. Its crystal structure contains two different perfluoridotitanate (IV) anion–cubic [Ti_8_F_36_]^4−^ octamers and a hexameric [Ti_6_F_28_]^4−^ anion. Unfortunately, it was not possible to accurately determine all cations in the crystal structure, but the proposed models of the anions are well refined. The [Ti_6_F_28_]^4−^ anion has a very unusual geometry (Figure 19). In the centre are two TiF_6_ octahedra that share a vertex. Attached to this pair is a TiF_6_ unit that shares a fluorine atom with each of the octahedra. There is also a chain of three TiF_6_ octahedra in which each octahedron at the end of the chain shares two vertices with two octahedra in the centre of the [Ti_6_F_28_]^4−^ anion.

### 2.13. [M_8_F_36_]^4−^ Anion (M = Ti, Mn)

A summary of the crystal data of the salts consisting of [M_8_F_36_]^4−^ anions (M = Ti, Mn) is given in Table 9.

**Figure 20:**
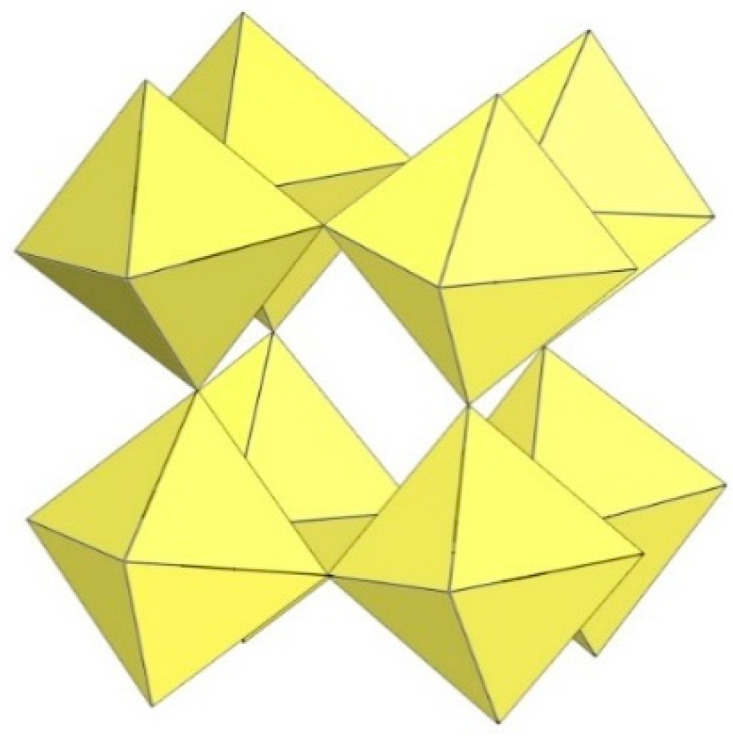
Octameric [Ti_8_F_36_]^4−^ anion in the crystal structures of K_4_[Ti_8_F_36_]·8HF, Rb_4_[Ti_8_F_36_]·6HF, and [H_5_O_2_]_4_[Ti_8_F_36_].

The [Ti_8_F_36_]^4−^ anion in K_4_Ti_8_F_36_·8HF [24], Rb_4_Ti_8_F_36_·6HF [24], and [H_5_O_2_]_4_[Ti_8_F_36_] [22] resembles a cube species consisting of eight TiF_6_ octahedra, with the eight titanium atoms located at the vertices of a cube (Figure 20). Each of the TiF_6_ octahedra shares three fluorine atoms (in the fac position) with three neighbouring TiF_6_ octahedra. In K_4_Ti_8_F_36_·8HF, the Ti–F_t_ bond lengths are 1.755(2)–1.801(2) Å and Ti–F_b_ 1.9239(19)–2.0139(19) Å, while in Rb_4_Ti_8_F_36_·6HF, the Ti–F_t_ bond distances are 1.754(6)–1.783(6) Å and Ti–F_b_ 1.939(7)–2.005(6) Å [24]. Both sets of distances are consistent with those previously observed in various fluoride–titanate (IV) compounds. The crystal structure of the compound [H_5_O_2_]_4_[Ti_8_F_36_] consists of octameric [Ti_8_F_36_]^4−^ anions (Figure 20) and asymmetric [H_5_O_2_]^+^ cations. The former have a similar geometry (Ti–F_t_ bonds with lengths of 1.757(1), 1.780(1), 1.784(1) Å and five Ti–F_b_ bonds with lengths of 1.956(1), 2 × 1.964(1), and 2 × 1.9738(4) Å) [22] as in the crystal structures of K_4_[Ti_8_F_36_]·8HF and Rb_4_[Ti_8_F_36_]·6HF [24].

**Figure 21:**
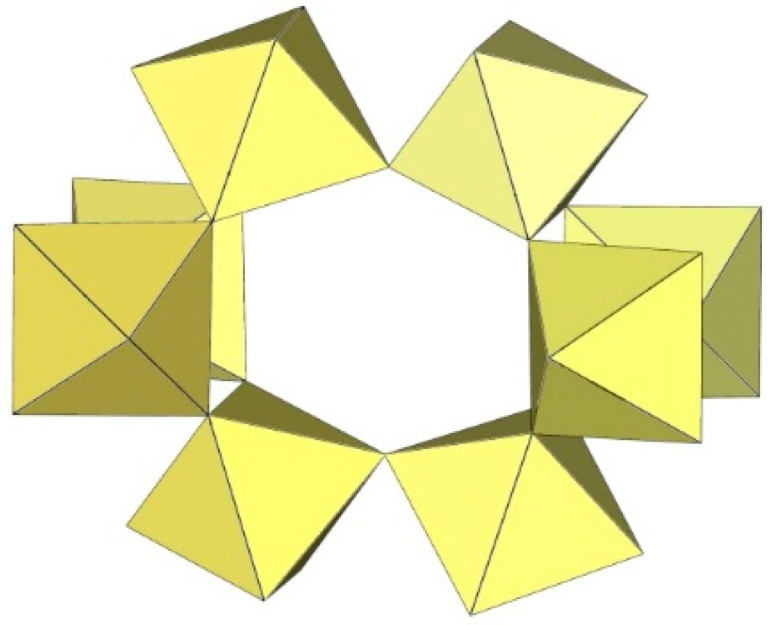
Octameric [Mn_8_F_36_]^4−^ anion in the crystal structure of [XeF_5_]_4_[Mn_8_F_36_].

The geometry of the [Mn_8_F_36_]^4−^ anion (Figure 21) in [XeF_5_]_4_[Mn_8_F_36_] [25] is completely different from that of the [Ti_8_F_36_]^4−^ anion. In [XeF_5_]_4_[Mn_8_F_36_], each MnF_6_ octahedron of [Mn_8_F_36_]^4−^ shares three fluorine atoms (in fac position) with three neighbouring MnF_6_ octahedra, resulting in a ring-shaped [Mn_8_F_36_]^4−^ geometry. Each [Mn_8_F_36_]^4−^ anion forms secondary F···Xe contacts with six [XeF_5_]^+^ cations. The Mn–F bond distances can be divided into three groups. The Mn–F(···Xe), where F is involved in secondary contacts with [XeF_5_]^+^ cations, are longer (1.740(2)–1.765(2) Å) than Mn–F_t_ bonds (F_t_ = terminal fluorine atoms without further interactions; 1.710(2)–1.717(2) Å) but shorter than the Mn–F_b_(–Mn) bond distances (F_b_ = fluorine atoms bridging two Mn atoms; 1.8498(19)–1.9529(19) Å) [25].

### 2.14. [M_10_F_45_]^5−^ Anion (M = Ti)

A summary of the crystal data of the salt consisting of [M_10_F_45_]^5−^ anions (M = Ti) is given in Table 10.

**Figure 22:**
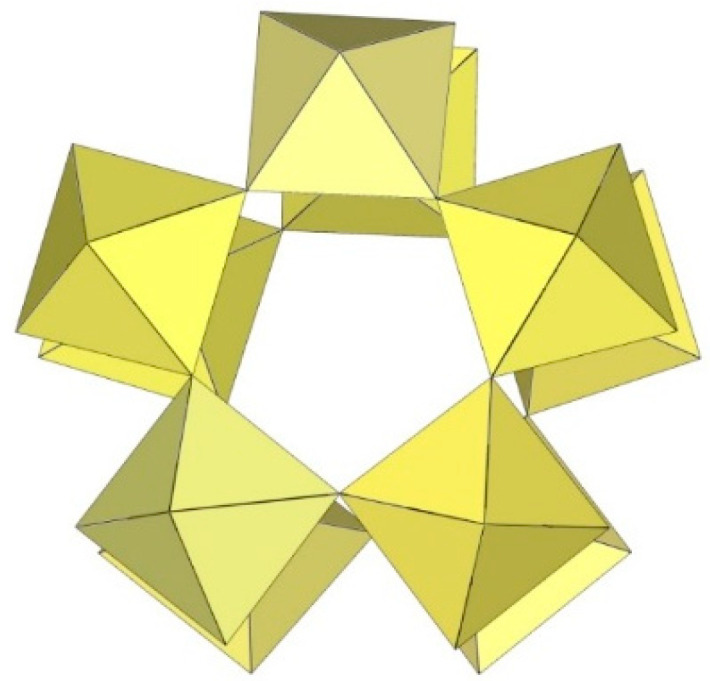
Decameric [Ti_10_F_45_]^5−^ anion in the crystal structure of [XeF_5_]_5_[Ti_10_F_45_].

The crystal structure determination of [XeF_5_]_5_[Ti_10_F_45_] reveals the largest known discrete perfluometallate (IV) anion [Ti_10_F_45_]^5−^ (Figure 22) [26]. [XeF_5_]_5_[Ti_10_F_45_] crystallises in two crystal modifications at low (α-phase, 150 K) and ambient (β-phase, 296 K) temperatures. The crystal structure of β-[XeF_5_]_5_[Ti_10_F_45_] consists of [XeF_5_]^+^ cations and discrete decameric [Ti_10_F_45_]^5−^ anions composed of ten TiF_6_ octahedral units. Each of the ten TiF_6_ octahedra shares three fac-vertices with neighbouring TiF_6_ units, resulting in a double ring-like geometry of the [Ti_10_F_45_]^5−^ anion. The bond lengths of Ti–F_t_ and Ti–F_b_ are in the range of 1.728(7)–1.823(6) Å and 1.916(6)–2.006(6) Å, respectively [26]. The low-temperature phase α-[XeF_5_]_5_[Ti_10_F_45_] is monoclinic. The main difference between the α- and β-[XeF_5_]_5_[Ti_10_F_45_] phases is that the [XeF_5_]^+^ cations in the α-phase are fully ordered, whereas one of the three crystallographically unique [XeF_5_]^+^ cations in the β-phase is two-fold disordered.

## 3. Polymeric Chain-like ([MF_5_]^−^)_∞_ Anion (M = Ti, V, Cr, Mn, Ge, Sn, Pb)

Polymeric ([MF_5_]^−^)_∞_ anions consist of single chains of MF_6_ octahedra connected via cis- or trans-vertices or both as in (XeF_5_CrF_5_)_4_·XeF_4_.

### 3.1. Trans-([MF_5_]^−^)_∞_ Anion (M = Ge, Cr)

A summary of the crystal data of the salts consisting of trans-([MF_5_]^−^)_∞_ anions (M = Ge, Cr) is given in Table 11.

**Figure 23:**
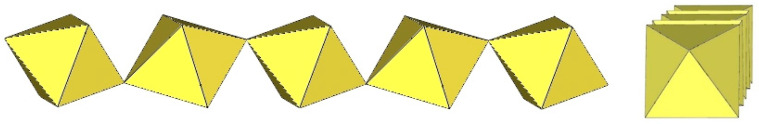
Polymeric trans-([GeF_5_]^−^)_∞_ chain in the crystal structure of XeF_5_GeF_5_.

The crystal structure of XeF_5_GeF_5_ is a rare case in which M^IV^F_6_ octahedra share their F atoms in trans position to form infinite ([MF_5_]^−^)_∞_ chain-like anions (Figure 23) [27]. The coordination around each Ge atom is an elongated octahedron of fluorine atoms. The Ge–F_b_–Ge angle is equal to 140.70(20)° [27]. Viewed along the GeF_5_ chain, all F_t_ are in eclipsed positions. All Ge–F_t_ distances within the square plane are equal at 1.745(2) Å, and the Ge–F_b_ distance is 1.890(1) Å [27].

**Figure 24:**
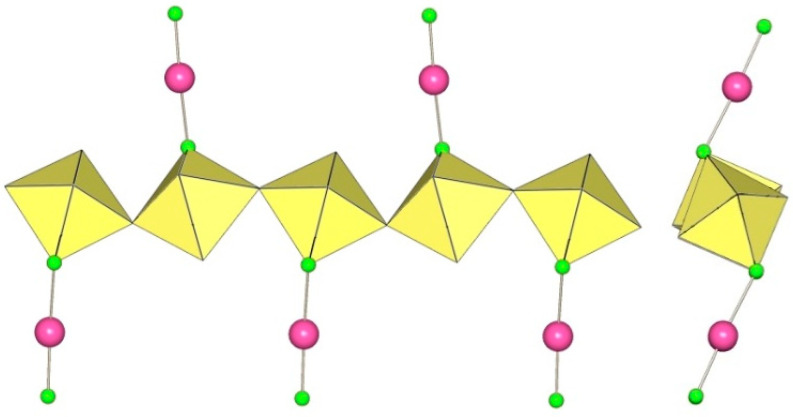
Polymeric trans-([CrF_5_]^−^)_∞_ chain in the crystal structure of XeF_2_·CrF_4_.

The Xe–F bond lengths in XeF_2_·CrF_4_ indicate that XeF_2_ is at the beginning of its ionization pathway (XeF_2_ → [XeF]^+^ + F^−^) [28]. Therefore, the formulation of the compound as the adduct XeF_2_·CrF_4_ is more suitable than the ionic formulation [XeF]^+^[CrF_5_]^−^. The structure of XeF_2_·CrF_4_ consists of an infinite chain of CrF_6_ octahedra sharing trans-vertices (Figure 24). For each CrF_6_ octahedron, one F atom is provided by a XeF_2_ molecule. The CrF_6_ unit consists of three F_t_ (1.71(2)–1.75(2) Å) and three F_b_ (1.88(2)–2.00(2) Å) atoms [28]. The Cr–F_b_–Cr angle is 147.3(8)° [28]. Viewed along the ([CrF_5_]^−^)_∞_ chain, all F_t_ are in eclipsed positions.

### 3.2. Cis-([MF_5_]^−^)_∞_ Anions (M = Ti, V, Cr, Mn, Ge, Sn, Pb)

There are many more examples of polymeric ([MF_5_]^−^)_∞_ anions (M = Ti, V, Cr, Mn, Ge, Sn, Pb) in which MF_6_ octahedra share F atoms in the cis position, especially in the case of titanium. The different tilting of the MF_6_ octahedra in the chains leads to small differences in their geometry. A summary of the crystal data of the salts consisting of cis-([MF_5_]^−^)_∞_ anions (M = Ti, V, Cr, Mn, Ge, Sn, Pb) is given in Table 12.

**Figure 25:**
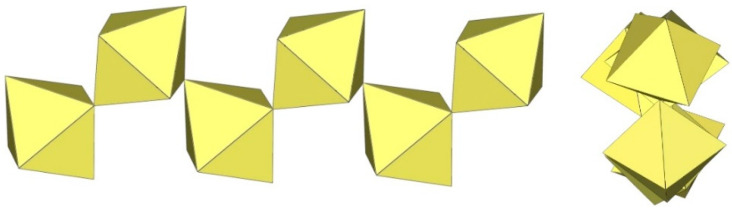
Polymeric cis-([TiF_5_]^−^)_∞_ chain in the crystal structure of H_3_OTiF_5_.

H_3_OTiF_5_ crystallizes in the monoclinic space group *C*2/*c* (Table 12) [29]. The Ti–F_b_–Ti angle is 146.56° (Figure 25) [29].

**Figure 26:**
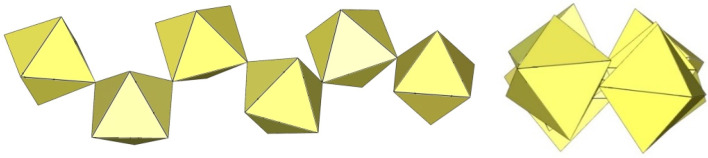
Polymeric cis-([TiF_5_]^−^)_∞_ chain in the crystal structure of NH_4_TiF_5_.

NH_4_TiF_5_ crystallizes in the monoclinic space group P2_1_/n (Table 12) [30]. The Ti–F_b_–Ti angles are in the range 155.09–164.11° (Figure 26) [30].

**Figure 27:**
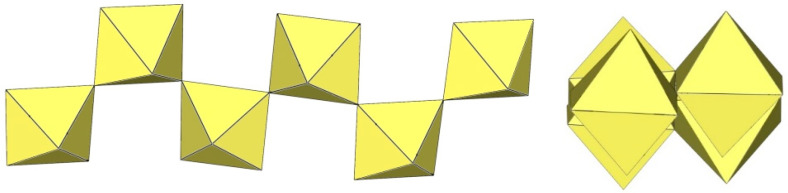
Polymeric cis-([TiF_5_]^−^)_∞_ chain in the crystal structure of NaTiF_5_·HF.

NaTiF_5_·HF crystallizes in the monoclinic space group C/2c (Table 12) [31]. The compound is composed of infinite single chains of ([TiF_5_]^−^)_∞_ anions (Figure 27), Na^+^ cations, and coordinated HF molecules. The Ti–F_t_ bond lengths range from 1.769(2) Å to 1.888(2) Å and are shorter than the Ti–F_b_ bond lengths, which are 1.965(1) Å and 2.009(1) Å, respectively [31]. The observed Ti–F_b_–Ti angles are 180.0° and 154.5(2)° [31].

**Figure 28:**
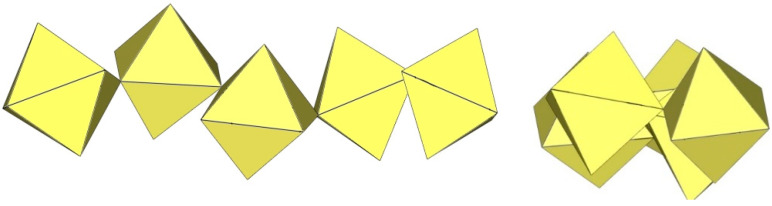
Polymeric cis-([TiF_5_]^−^)_∞_ chain in the crystal structure of KTiF_5_.

KTiF_5_ crystallizes in the monoclinic space group *C*2/*c* (Table 12) [31]. The fluorine atoms are partially disordered (Figure 28).

**Figure 29:**
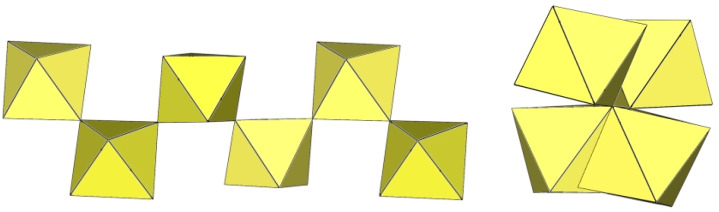
Polymeric cis-([TiF_5_]^−^)_∞_ chain in the crystal structures of KTiF_5_·HF and RbTiF_5_·HF.

The crystal structures of KTiF_5_·HF and RbTiF_5_·HF are isotypic [31]. They crystallize in the monoclinic space group *C*2/*c* (Table 12). The bond lengths between Ti and F_t_ atoms are in the range 1.795(3)–1.859(3) Å for K[TiF_5_]·HF and 1.791(4)–1.862(4) Å for Rb[TiF_5_]·HF [31]. The longest Ti–F bond lengths are between Ti atoms and F_b_ atoms bridging two octahedra (1.9605(7) Å and 1.9630(14) Å in KTiF_5_·HF; 1.9639(12) Å and 1.968(2) Å in Rb[TiF_5_]·HF) [31]. In contrast to the ([TiF_5_]^−^)_∞_ anions described in [H_3_O][TiF_5_], [NH_4_][TiF_5_], and Na[TiF_5_]·HF, the chains in K[TiF_5_]·HF and Rb[TiF_5_]·HF have a significantly different conformation (Figure 29). Each TiF_6_ octahedron is connected to two neighbouring TiF_6_ units via bridging F atoms located in cis positions of the single octahedron, with the observed Ti–F_b_–Ti angles being 180.0° (K, Rb salt), 148.5(2)° (K salt), and 147.2(3)° (Rb-salt) [31].

**Figure 30:**
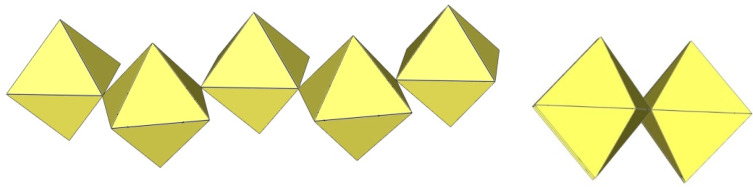
Polymeric cis-([TiF_5_]^−^)_∞_ chain in the crystal structure of CsTiF_5_.

CsTiF_5_ crystallizes in the orthorhombic space group Pnma (Table 12) [31]. Each of the terminal fluorine atoms is disordered over two crystallographic positions (Figure 30). The observed Ti–F_t_ bond lengths range from 1.687(11) Å to 1.904(6) Å, and the Ti–F_b_ bond lengths are 1.972(2) Å and 1.982(2) Å [31]. The Raman spectrum of Cs[TiF_5_] recorded on a single crystal is identical to the previously reported Raman spectrum of “Cs_2_[Ti_2_F_10_]”, which was claimed to consist of discrete [Ti_2_F_10_]^2−^ anions [31]. These results show that the previously reported Cs_2_[Ti_2_F_10_] is, in fact, Cs[TiF_5_].

**Figure 31:**
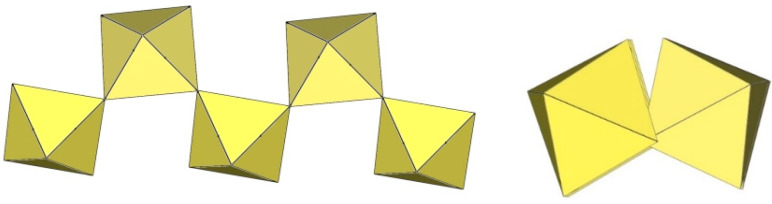
Polymeric cis-([TiF_5_]^−^)_∞_ chain in the crystal structure of [enH_2_](TiF_5_)_2_ (en = ethane-1,2-diamine).

[enH_2_](TiF_5_)_2_ (en = ethane-1,2-diamine) crystallizes in the monoclinic space group P2_1_/c (Table 12, Figure 31) [32]. The bond distances between Ti and F_t_ are between 1.780(1) and 1.850(2) Å, and the bond distances between Ti and F_b_ are between 2.023(1) and 2.028(1) Å [32]. All Ti–F_b_–Ti angles in the crystal structure of [enH_2_][TiF_5_]_2_ are equivalent and correspond to 138.31(7)° [32].

**Figure 32:**
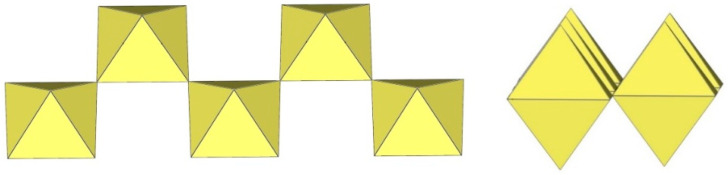
Polymeric cis-([VF_5_]^−^)_∞_ chain in the crystal structure of [H_3_N(CH_2_)_2_NH_2_][VF_5_].

The crystal structure of [H_3_N(CH_2_)_2_NH_2_][VF_5_] is a rare example of a structurally characterized V(IV) fluoride compound that does not consist only of [VF_6_]^2−^ anions [33]. The anionic part is composed of polymeric infinite ([VF_5_]^−^)_∞_ chains (Figure 32). The chain consists of V(IV) octahedra that share cis-vertices to form a zig-zag profile. The V–F distances are 1.838(3) and 2.132(5) Å for the terminal and bridging fluorides, respectively [33]. The presence of V(IV) was confirmed by charge-balance considerations and b magnetic studies. The V–F_b_–V angle is linear (180°) [33]. The F_t_ atoms of every second octahedron are in an eclipsed conformation (Figure 32).

**Figure 33:**
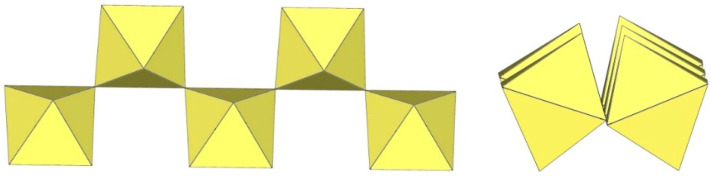
Polymeric cis-([CrF_5_]^−^)_∞_ chain in the crystal structure of RbCrF_5_.

The crystal structure of RbCrF_5_ (KCrF_5_ appears to be isotypic) crystallizes in the orthorhombic space group Pmc2_1_ (Table 12) [15]. The Cr–F_t_ bond distances in the cis-([CrF_5_]^−^)_∞_ chain (Figure 33) are between 1.780(1) and 1.850(2) Å and Cr–F_b_ between 2.023(1)–2.028(1) Å [15]. The Cri–F_b_–Cr angles are equal to 149.4(3)° [15]. Distorted [CrF_6_] octahedra have four terminal fluorine atoms with Cr–F_t_ distances in the range of 1.743(8)–1.782(7) Å and two bridging fluorine atoms with Cr–F_b_ distances of 1.945(5) and 1.948(5) Å [15].

**Figure 34:**
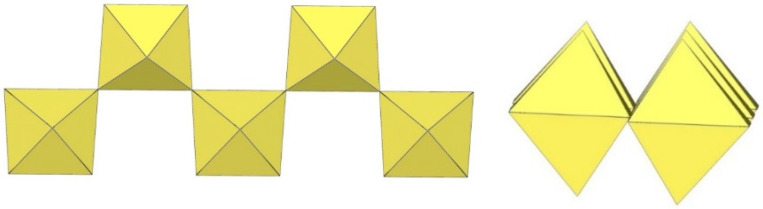
Polymeric cis-([CrF_5_]^−^)_∞_ chain in the crystal structure of CsCrF_5_.

CsCrF_5_ crystallizes in the orthorhombic space group Pnma (Table 12) [15]. The main feature of the CsCrF_5_ structure is also a ([CrF_5_]^−^)_∞_ chain of distorted [CrF_6_] octahedra connected by common cis-vertices (Figure 34). While the Cr–F_b_–Cr angle in the Rb salt is bent, the corresponding angle in the Cs salt is linear (180°) [15]. The magnetic measurements show an antiferromagnetic interaction between the magnetic moments of Cr(IV) in ACrF_5_ due to the coupling through Cr–F_b_–Cr bridges [38]. In ACrF_5_ (A = K, Rb), a weak ferromagnetic ground state was observed below T_c_ ~ 6 K, which can be explained as canted antiferromagnetism in correlation with the crystal structures of these two compounds.

**Figure 35:**
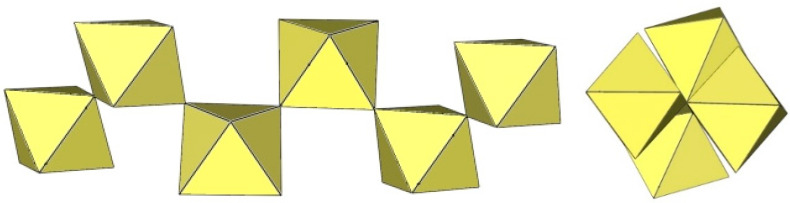
Polymeric cis-([GeF_5_]^−^)_∞_ chain in the crystal structure of O_2_GeF_5_·HF.

In contrast to XeF_5_GeF_5_ with a trans-shared GeF_6_ octahedra [27], the anion in the crystal structure of O_2_GeF_5_·HF (monoclinic space group I2/a, Table 12) consists of infinite ([GeF_5_]^−^)_∞_ chains of GeF_6_ octahedra sharing cis-vertices (Figure 35) [34]. The HF molecules and O_2_^+^ cations are located between the chains. The Ge–F_t_ bond lengths range from 1.729(2) Å to 1.7545(19) Å and are shorter than Ge–F_b_ (1.8817(3) Å and 1.8934(9) Å). There are alternating Ge–F_b_–Ge angles of 180.0° and 140.04(13)° [34].

**Figure 36:**
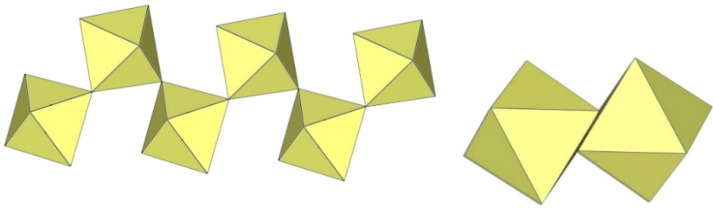
Polymeric cis-([SnF_5_]^−^)_∞_ chain in the crystal structure of ClO_2_SnF_5_.

The crystal structure of ClO_2_SnF_5_ is a rare example of a structurally determined Sn salt with a polymeric pentafluoridostannate (IV) anion, ([SnF_5_]^−^)_∞_ (Figure 36) [35]. The previous reports on the structures of the [SnF_5_]^−^ anions in the [NF_4_]^+^, [N_2_F_3_]^+^, and [N_5_]^+^ salts were based only on vibrational and/or ^19^F NMR spectroscopy in the solid state and in solution, respectively [39,40,41,42]. The ([SnF_5_]^−^)_∞_ anion is a linear zig-zag chain consisting of cis-bridged [SnF_6_] polyhedra. The Sn–F bond length of ClO_2_SnF_5_ is in the range of 1.9047(13)–2.0627(13) Å [35]. The Sn–F_b_–Sn angle is equal to 143.16° [35].

**Figure 37:**
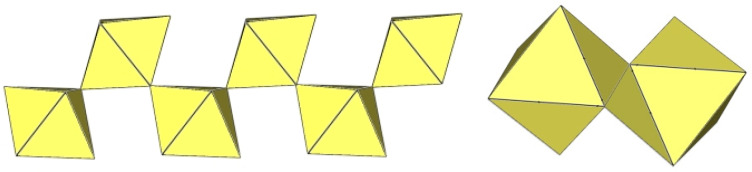
Polymeric cis-([MF_5_]^−^)_∞_ chain (M = Sn, Pb) in the crystal structure of ClOF_2_MF_5_ (M = Sn, Pb).

The crystal structures of ClOF_2_SnF_5_ and ClOF_2_PbF_5_ are isotypic (Table 12) [36]. The polymeric ([SnF_5_]^−^)_n_ anion has a similar chain-like geometry (Sn–F: 1.901(6)–2.061(5) Å and Sn–Fb–Sn = 143.31°) as in ClO_2_SnF_5_. Apart from the known [PbF_6_]^2−^ anion, the polymeric ([PbF_5_]^−^)_∞_ anion (Figure 37) is the only known example of a fluoridoplumbate (IV) anion. The Pb–F bond lengths are in the range of 1.979(3)–2.156(3) Å and the Pb–F_b_–Pb angle is 140.74° [36].

**Figure 38:**
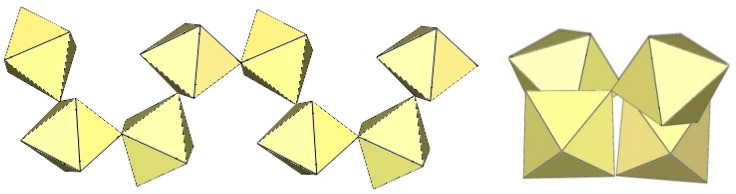
Polymeric cis-([CrF_5_]^−^)_∞_ chain in the crystal structure of XeF_5_CrF_5_.

The crystal structure of XeF_5_CrF_5_ [37] (XeF_5_TiF_5_ appears to be isotypic [26]) crystallizes in the orthorhombic space Pbca (Table 12; Figure 38). The ([CrF_5_]^−^)_∞_ chain also consists of CrF_6_ octahedra that share cis-vertices to form a zig-zag profile. However, the chain geometry differs from the polymeric ([MF_5_]^−^)_∞_ chain structures of [H_3_N(CH_2_)_2_NH_2_][VF_5_] [33], RbCrF_5_ [15], CsCrF_5_ [15], O_2_GeF_5_·HF [34], ClO_2_SnF_5_ [35], and ClOF_2_MF_5_ (M = Sn, Pb) [36]. The Cr–F bond lengths range from 1.675(11) Å to 1.971(10) Å) [37]. The Cr–F_b_–Cr bridges are kinked with angles of 144.8(5) and 147.4(6)° [37].

XeF_5_TiF_5_ crystallizes in the orthorhombic space group Pbca (Table 12) [26] and is most likely isotypic with XeF_5_CrF_5_ [37].

**Figure 39:**
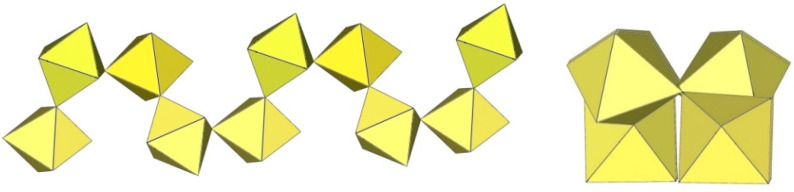
Polymeric cis-[(MnF_5_]^−^)_∞_ chain in the crystal structure of XeF_5_MnF_5_.

Single crystals of red [XeF_5_][MnF_5_] were grown in the form of very thin and fragile plates [25], which resulted in poor quality of the collected X-ray data. An attempt to improve the crystal structure of [XeF_5_][MnF_5_] by synchrotron X-ray powder diffraction (SXRD) resulted I a monoclinic unit cell (Table 12) [25]. According to the SXRD analysis, the crystal structure of [XeF_5_][MnF_5_] (Figure 39) is slightly different from [XeF_5_][CrF_5_] and [XeF_5_][TiF_5_] (Table 12). XeF_5_MnF_5_ is paramagnetic in the temperature range of 296–200 K, with a Curie constant of C = 1.87 emu K mol–1 (μ_eff_ = 3.87 μB) and a Curie–Weiss temperature of θ = −9.3 K. Below 100 K, there is weak antiferromagnetic coupling between the Mn^IV^ ions, with a coupling constant of J = −1.3 cm^−1^ [25].

**Figure 40:**
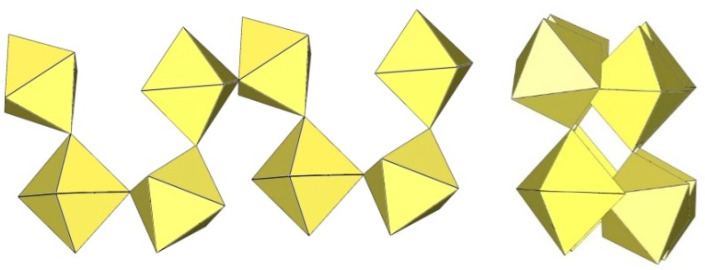
Polymeric cis-(TiF_5_]^−^)_∞_ chain in the crystal structure of [C(NH_2_)_3_]_4_(H_3_O)_4_[Ti_4_F_20_][TiF_5_]_4_.

The crystal structure determination of [C(NH_2_)_3_]_4_(H_3_O)_4_[Ti_4_F_20_][TiF_5_]_4_ provided the first example of a perfluoridotitanate (IV) compound with two different perfluoridotitanate (IV) anions in the same salt [22]. The latter appears as a crenelated chain (Figure 40), which is also observed in XeF_5_MF_5_ (M = Ti [26], Cr [37], Mn [25]). The Ti–F bond lengths are typical for poly[perfluoridotitanate (IV)] compounds and range from 1.763(1) to 1.877(1) A and from 1.964(1) to 2.004(1) A for the Ti–F_t_ and Ti–F_b_ bonds, respectively [22]. The Ti–F_b_–Ti angles are 148.48(7) and 157.07(7)° [22].

**Figure 41:**
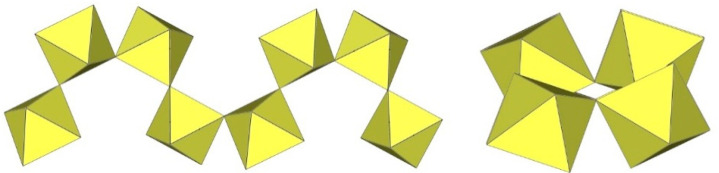
Polymeric cis-(GeF_5_]^−^)_∞_ chain in the crystal structure of ClO_2_GeF_5_.

The crystal structure of ClO_2_GeF_5_ consists of infinite ([GeF_5_]^−^)_∞_ chains (Figure 41) [27]. However, their geometry differs from the geometry of the ([GeF_5_]^−^)_∞_ chains in O_2_GeF_5_·HF [34], where the GeF_6_ octahedra also share common cis-vertices. The chains in the former salt are crenelated and not linear as in the case of O_2_GeF_5_·HF. The Ge–F_t_ bond lengths range from 1.73 Å to 1.78 Å and are shorter than the Ge–F_b_ bond lengths of 1.887(1) Å [27]. The Ge–F_b_–Ge angles are 148.1° and 143.4° [27].

### 3.3. Cis- and Trans-([MF_5_]^−^)_n_ Anions (M = Cr)

A summary of the crystal data of the salt consisting of cis- and trans-([MF_5_]^−^)_n_ anions (M = Cr) is given in Table 13.

**Figure 42:**
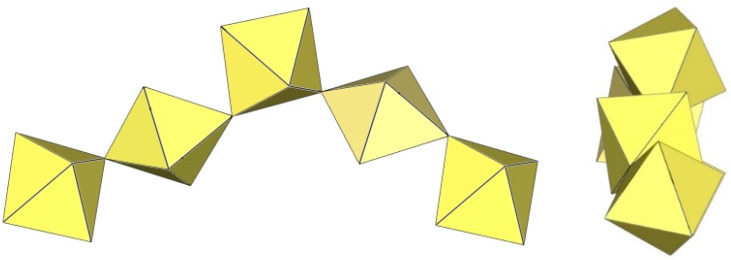
Polymeric cis-and trans-([CrF_5_]^−^)_∞_ chain in the crystal structure of (XeF_5_CrF_5_)_4_·XeF_4_.

The crystal structure of (XeF_5_CrF_5_)_4_·XeF_4_ consists of infinite chains of distorted CrF_6_ octahedra sharing alternating trans- and cis-vertices (Figure 42) and is the only example of its kind [28]. Cr–F_t_ bond lengths range from 1.701(8) Å to 1.895(7) Å and Cr–F_b_ from 1.8890(6) Å to 1.961(7) Å [28]. The Cr–F_b_–Cr angles are 136.6(4) and 142.3(4)° [28].

## 4. Polymeric Double Chain-like ([M_2_F_9_]^−^)_∞_ Anions (M = Ti, Mn, Sn)

A summary of the crystal data of the salts consisting of double chain-like ([M_2_F_9_]^−^)_∞_ anions (M = Ti, Mn, Sn) is given in Table 14.

**Figure 43:**
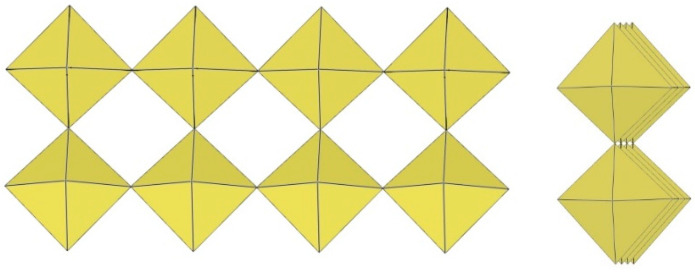
Polymeric chain-like ([Sn_2_F_9_]^−^)_∞_ anion in the crystal structure of α-O_2_Sn_2_F_9_.

The polymeric ([Sn_2_F_9_]^−^)_∞_ anion in α-O_2_Sn_2_F_9_ consists of two parallel, infinite chains composed of SnF_6_ octahedra, with each SnF_6_ octahedron of one chain connected to a SnF_6_ octahedron of the second chain through a common fluorine vertex (Figure 43) [34]. The Sn–F_b_–Sn angles within each chain are equal to 170.7(2)°, and the angles at which the Sn atoms belong to two neighbouring chains are linear (Sn–F_b_–Sn = 180°) [34]. The three Sn–F_b_ bonds between tin and the bridging fluorine atoms are longer (2.0303(3) Å–2.0374(4) Å) than the three Sn–F_t_ bonds between tin and the terminal fluorine atoms (1.898(2) Å–1.909(4) Å) [34]. The negative charge of the ([Sn_2_F_9_]^−^)_∞_ anions is compensated by partially disordered O_2_^+^ cations located between the chains.

**Figure 44:**
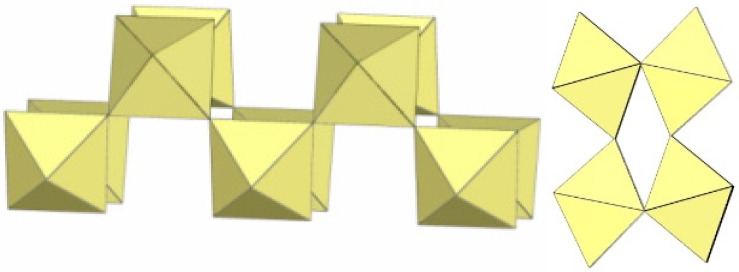
Polymeric chain-like ([Ti_2_F_9_]^−^)_∞_ anion in the crystal structure of α-[H_3_O][Ti_2_F_9_].

α-[H_3_O][Ti_2_F_9_] crystallizes at 100 K in the orthorhombic space group Pnma (Table 14) [6]. In contrast to the ([Sn_2_F_9_]^−^)_∞_ anion in α-O_2_Sn_2_F_9_ [34], the individual chains in the double chain ([Ti_2_F_9_]^−^)_∞_ anion are not linear (Figure 44). The Ti–F_b_–Ti angles within the individual single zig-zag chains are kinked with an angle of 166.4(2)°, and the Ti–F_b_–Ti angles, where the Ti atoms belong to two neighbouring chains, are 143.7(2)° [6].

**Figure 45:**
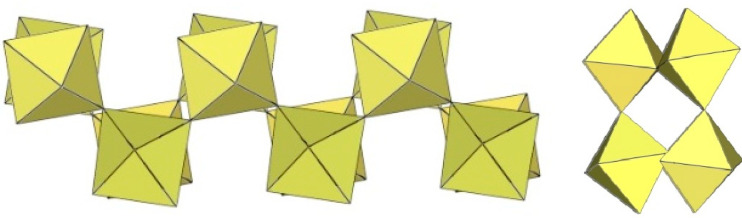
Polymeric chain-like ([Ti_2_F_9_]^−^)_∞_ anion in the crystal structure of β-[H_3_O][Ti_2_F_9_].

β-[H_3_O][Ti_2_F_9_] crystallizes at 150 K in the monoclinic space group P2_1_/c (Table 14) [22]. In contrast to the eclipsed structure of the double-chain ([Ti_2_F_9_]^−^)_∞_ anion in the orthorhombic modification α-[H_3_O][Ti_2_F_9_] (Figure 44) [6], the double-chain ([Ti_2_F_9_]^−^)_∞_ anion in the β-phase exhibits a gauche conformation of the TiF_6_ octahedra belonging to two parallel single chains (Figure 45).

**Figure 46:**
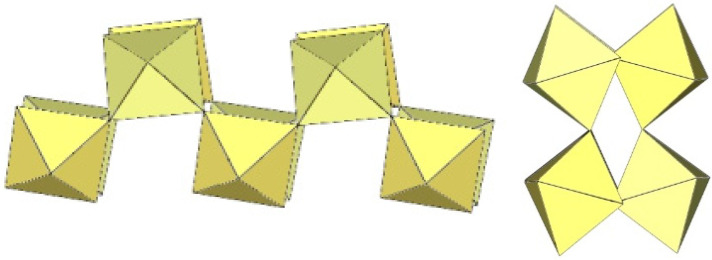
Polymeric chain-like ([Ti_2_F_9_]^−^)_∞_ anion in the crystal structure of NaTi_2_F_9_·HF.

The crystal structure of NaTi_2_F_9_·HF also consists of zig-zag ([Ti_2_F_9_]^−^)_∞_ double chains (Figure 46) [31]. The Ti–F_b_ bond lengths are in the range 1.963(2)–1.974(1) Å and the Ti–F_t_ bond lengths are in the range 1.768(2)–1.787(2) Å [31]. The Ti–F_b_–Ti angles within individual single chains correspond to 158.7(1)° [31]. The Ti–F_b_–Ti angles in which the Ti atoms belong to the two neighbouring single chains of the dimer are equal to 141.4(1)° [31]. The closest TiF_6_ octahedra belonging to two individual single chains are in an eclipsed conformation to each other. The Na[Ti_2_F_9_]·HF compound contains HF molecules. There are hydrogen bond interactions between the HF molecules and the polymeric ([Ti_2_F_9_]^−^)_∞_ anion.

**Figure 47:**
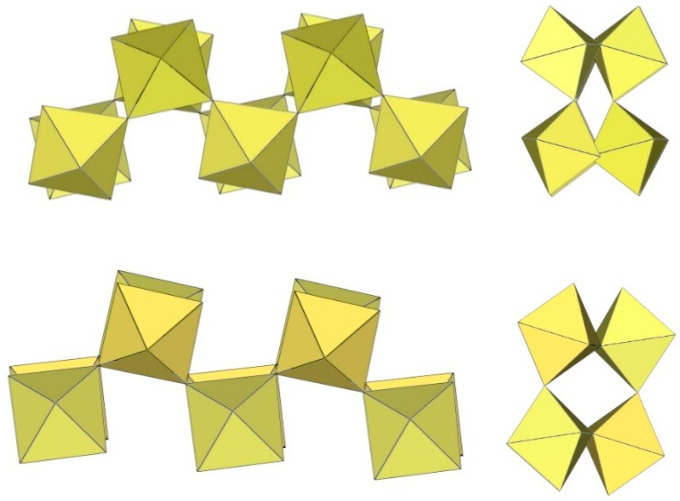
Polymeric chain-like ([Ti_2_F_9_]^−^)_∞_ anions in the crystal structure of RbTi_2_F_9_.

The single-crystal structure of Rb[Ti_2_F_9_] consists of an infinite ([Ti_2_F_9_]^−^)_∞_ anion in two different conformations (Figure 47) [31]. One ([Ti_2_F_9_]^−^)_∞_ anion has a gauche conformation of the TiF_6_ octahedral pairs belonging to the two single chains of the double chain, as in the anions in the crystal structures of β-H_3_OTi_2_F_9_ (Figure 45), while the second anion has an eclipsed conformation of these TiF_6_ octahedral pairs, similar to the anion in the crystal structures of α-[H_3_O][Ti_2_F_9_] (Figure 44).

**Figure 48:**
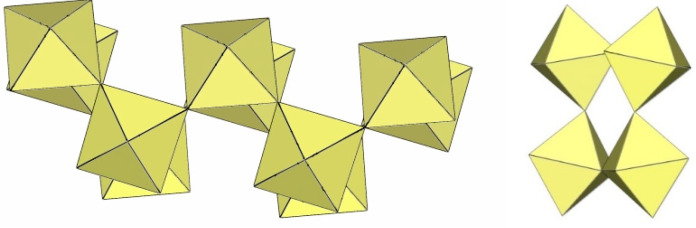
Polymeric chain-like ([Ti_2_F_9_]^−^)_∞_ anion in the crystal structure of CsTi_2_F_9_.

CsTi_2_F_9_ crystallizes in the monoclinic space group *C*2/*c* (Table 14, Figure 48) [6], where the Ti–F_b_–Ti angles within the individual zig-zag chains are kinked with an angle of 156.3(4)°. The Ti–F_b_–Ti angles where Ti atoms belong to two neighbouring chains are 149.3(6)° [6].

**Figure 49:**
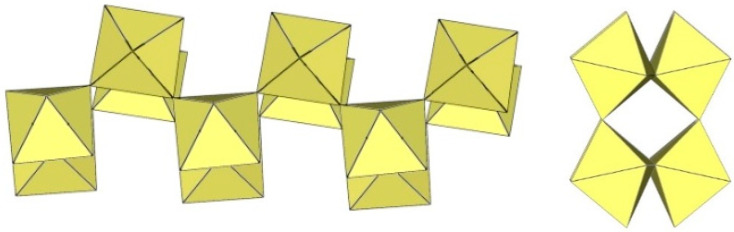
Polymeric chain-like ([Ti_2_F_9_]^−^)_∞_ anion in the crystal structure of α-[ImH][Ti_2_F_9_].

**Figure 50:**
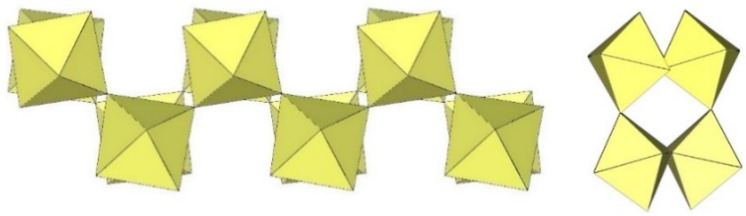
Polymeric chain-like ([Ti_2_F_9_]^−^)_∞_ anion in the crystal structure of β-[ImH][Ti_2_F_9_].

α-[ImH][Ti_2_F_9_] crystallizes at 200 K in the monoclinic space group P2_1_/a (Table 14), while β-[ImH][Ti_2_F_9_] is orthorhombic at 298 K (Table 14) [12]. The geometry of the ([Ti_2_F_9_]^−^)_∞_ anions in both structures (Figure 49 and Figure 50) show the same behaviour as in α- and β-[H_3_O][Ti_2_F_9_] (Figure 44 and Figure 45). In α-[ImH][Ti_2_F_9_], the double-chain ([Ti_2_F_9_]^−^)_∞_ anion exhibits an eclipsed conformation of TiF_6_ octahedra belonging to two parallel single chains (Figure 49), while this conformation in β-[ImH][Ti_2_F_9_] is gauche (Figure 50). The Ti–F_b_–Ti angles within the single zig-zag chains of the dimers are crystallographically equivalent in β-[ImH][Ti_2_F_9_] [152.1(1)°], while these angles are comparable in α-[ImH][Ti_2_F_9_] [149.54(8)° and 151.21(9)°] [12]. The Ti–F_b_–Ti angles in [ImH][Ti_2_F_9_], where the titanium atoms belong to two neighbouring chains, are the same, within ±3σ for the α-phase [162.27(8)°] and the β-phase [163.1(2)°] [12].

**Figure 51:**
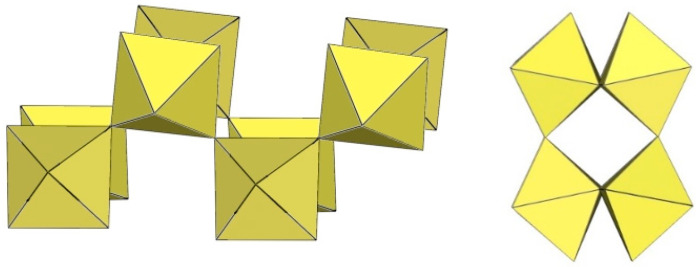
Polymeric chain-like ([Ti_2_F_9_]^−^)_∞_ anion in the crystal structure of [gvH][Ti_2_F_9_].

The geometry of the ([Ti_2_F_9_]^−^)_∞_ anion in [gvH][Ti_2_F_9_] (gv = guanidine) [22] is isostructural with the previous examples. It consists of TiF_6_ octahedra that share vertices at the fac position and form dimeric zig-zag chains (Figure 51). Each titanium atom is coordinated with three bridging and three terminal fluorine atoms, with Ti–F bonds ranging from 1.771(1) to 1.777(1) Å and from 1.9713(4) to 1.980(1) Å for Ti–F_t_ and Ti–F_b_, respectively [22]. The Ti–F_b_–Ti angles within each chain of dimers are equal to 155.01(7)° [22]. The Ti–F_b_–Ti angles where the titanium atoms belong to two neighbouring single chains of the dimer are equal to 163.5(1)° [22].

**Figure 52:**
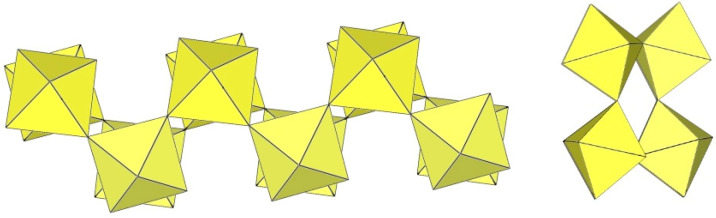
Polymeric chain-like ([Ti_2_F_9_]^−^)_∞_ anions in the crystal structure of [ClO_2_][Ti_2_F_9_].

[ClO_2_][Ti_2_F_9_] crystallizes in the monoclinic space group *C*2/*c* (Table 14, Figure 52) [35]. Each Ti atom is surrounded by six F atoms in the form of a distorted octahedron with Ti–F bond lengths of 1.776(2) to 1.980(2) Å [35].

**Figure 53:**
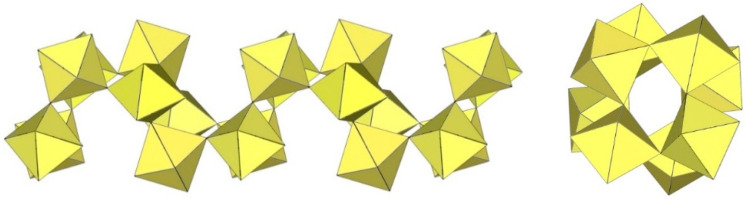
Polymeric chain-like ([Mn_2_F_9_]^−^)_∞_ anion in the crystal structure of [O_2_][Mn_2_F_9_].

O_2_Mn_2_F_9_ crystallizes at 148 K in the orthorhombic space group *C*2/*c* (Table 14) [43]. The crystal structure consists of ([Mn_2_F_9_]^−^)_∞_ anions with a unique geometry (Figure 53). The ([Mn_2_F_9_]^−^)_∞_ chains are crenelated and not linear as in other examples of ([M_2_F_9_]^−^)_∞_ (M = Sn, Ti) salts. It can be imagined to be composed of two single ([MnF_5_]^−^)_∞_ chains (as observed in [XeF_5_][MnF_5_] (Figure 39)), which additionally share some vertices to form a double ([Mn_2_F_9_]^−^)_∞_ chain.

## 5. Polymeric Column-like ([M_3_F_13_]^−^)_∞_, ([M_4_F_19_]^3−^)_∞_, ([M_7_F_30_]^2−^)_∞_ and ([M_9_F_38_]^2−^)_n_ Anions (M = Ti)

A summary of the crystal data of the salts consisting of polymeric column-like ([M_3_F_13_]^−^)_∞_, ([M_4_F_19_]^3−^)_∞_, ([M_7_F_30_]^2−^)_∞_, and ([M_9_F_38_]^2−^)_n_ anions (M = Ti) is given in Table 15.

**Figure 54:**
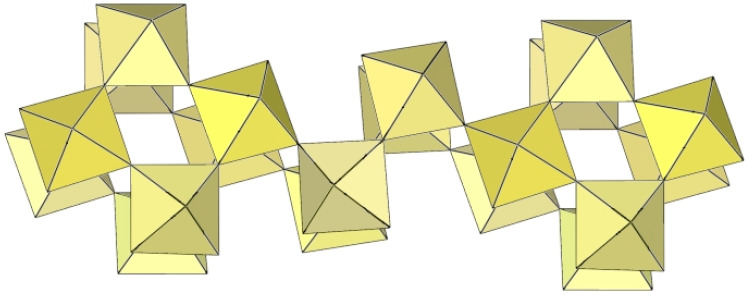
Polymeric column-like ([Ti_2_F_13_]^−^)_∞_ anion in the crystal structure of [XeF_5_][Ti_3_F_13_].

In [XeF_5_][Ti_3_F_13_], the anionic part consists of tetrameric Ti_4_F_20_ and octameric Ti_8_F_36_ units that share vertices and are alternatively connected to form ([Ti_3_F_13_]^−^)_∞_ columns (Figure 54) [26]. The negative charge of the anions is balanced by [XeF_5_]^+^ countercations interacting via secondary Xe···F bonds. The Ti–F_t_ bond lengths range from 1.728(5) to 1.813(5) Ǻ and are significantly shorter than the Ti–F_b_ bonds (1.942(4)–2.049(5) Å) [26].

**Figure 55:**
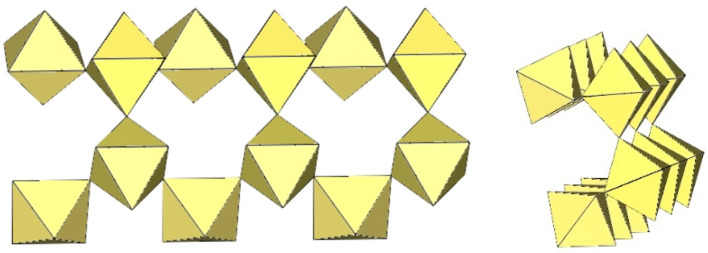
Polymeric column-like ([Ti_4_F_19_]^3−^)_∞_ anion in the crystal structure of Cs_3_[Ti_4_F_19_].

The polymeric ([Ti_4_F_19_]^3−^)_∞_ anion in Cs_3_[Ti_4_F_19_] consists of two zig-zag chains composed of TiF_6_ units (Figure 55) [31]. In contrast to the polymeric ([Ti_2_F_9_]^−^)_∞_ anion, the polymeric ([Ti_4_F_19_]^3−^)_∞_ anion lacks a second link between the TiF_6_ unit of one chain and the TiF_6_ unit of the second chain. The length distribution of the Ti–F_t_ and Ti–F_b_ bonds is in the range of 1.768(3)–1.833(4) Å and 1.958(3)–2.006(3) Å, respectively [31]. The Ti–F_b_–Ti angles within the zig-zag single chain are 155.7(2)°, and the Ti–F_b_–Ti angles between two single chains are 151.6(3)° [31].

Both the crystal structure of Cs_3_[Ti_4_F_19_] [31] and that of [XeF_5_]_3_[Ti_4_F_19_] [21] contain the anion, which can be expressed by the general formula [Ti_4_F_19_]^3−^. However, the [Ti_4_F_19_]^3−^ in [XeF_5_]_3_[Ti_4_F_19_] is an oligomeric species, whereas the [Ti_4_F_19_]^3−^ in Cs_3_[Ti_4_F_19_] is polymeric. Although many fluoridotmetallate (IV) anions are known, this is a rare case where two different geometries have been structurally determined for the same general formula of the anion.

**Figure 56:**
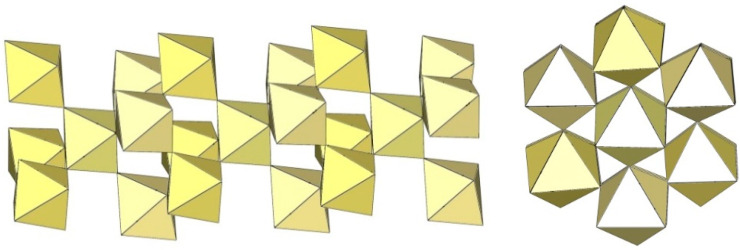
Polymeric column-like ([Ti_7_F_30_]^2−^)_∞_ anion in the crystal structure of (O_2_)_2_[Ti_7_F_30_].

The crystal structure of (O_2_)_2_ [Ti_7_F_30_] consists of column-like ([Ti_7_F_30_]^2−^)_∞_ anions (Figure 56) [44]. The structure of the ([Ti_7_F_30_]^2−^)_∞_ anion is comprised of cubic units of eight TiF_6_ octahedra, with two TiF_6_ units in opposite corners of the cube sharing vertices with neighbouring cubes. In this way, the Ti atoms common to the neighbouring cubes are coordinated by six bridging fluorine atoms, while the other Ti atoms are coordinated by three F_b_ and three F_t_ atoms. The negative charge of the anions is compensated by O_2_^+^ cations located between the ([Ti_7_F_30_]^2−^)_∞_ columns.

**Figure 57:**
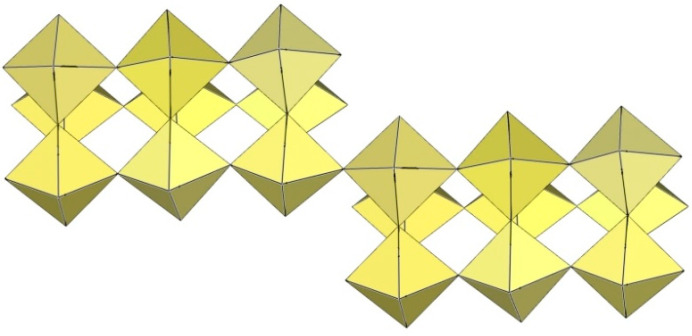
Polymeric column-like ([Ti_9_F_38_]^2−^)_∞_ anion in the crystal structure of [XeF]_2_[Ti_9_F_38_].

The crystal structure of [XeF]_2_[Ti_9_F_38_] consists of column-like ([Ti_9_F_38_]^2−^)_∞_ anions (Figure 57) [45]. Trimeric rings of TiF_6_ octahedra are linked to form trigonal prismatic Ti_9_F_39_ units, which are additionally connected by single fluorine bridges and form column-like ([Ti_9_F_38_]^2−^)_∞_ anions.

## 6. Polymeric *Layered* ([M_8_F_33_]^−^)_∞_ and ([M_2_F_9_]^−^)_∞_ *Anions* (M = Ti, Cr)

A summary of the crystal data of the salts consisting of layered ([M_8_F_33_]^−^)_∞_ and ([M_2_F_9_]^−^)_∞_ anions (M = Ti, Cr) is given in Table 16.

The infinite two-dimensional (2-D) arrangement of poly[perfluoridometallate (IV)] anions is observed in the case of the ([Ti_8_F_33_]^−^)_∞_ anion characterized in CsTi_8_F_33_ [46] and [Xe_2_F_3_][Ti_8_F_33_] [45]. In both cases, the ([Ti_8_F_33_]^−^)_∞_ anion represents a layered structure with different structural motifs.

**Figure 58:**
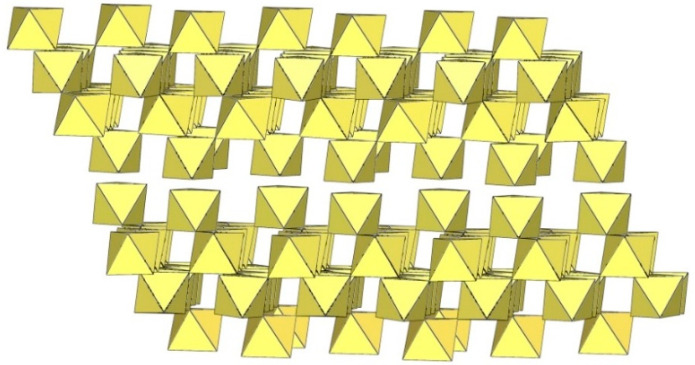
Packing of polymeric anionic layers ([Ti_8_F_33_]^−^)_∞_ in the crystal structure of CsTi_8_F_3_.

In CsTi_8_F_33_, two Ti atoms are coordinated by three bridging and three terminal fluorine atoms, while the other two are coordinated by four bridging and two terminal fluorine atoms, ultimately leading to a 2-D framework (Figure 58) [46].

**Figure 59:**
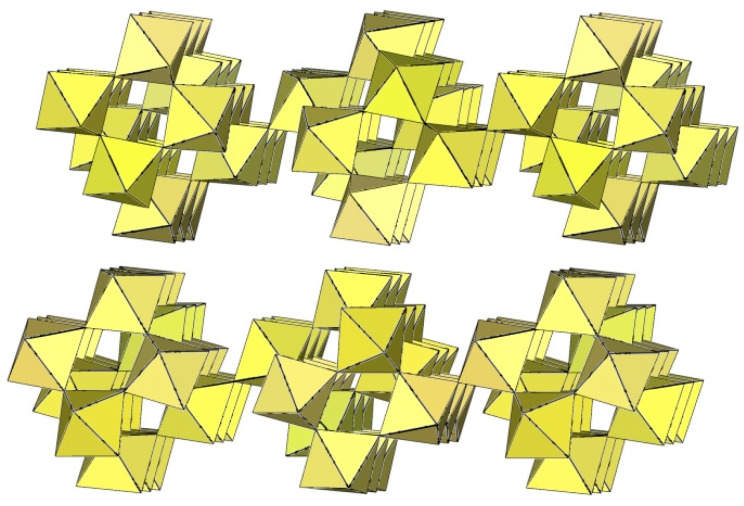
Packing of polymeric anionic layers ([Ti_8_F_33_]^−^)_∞_ in the crystal structure of [Xe_2_F_3_][Ti_8_F_33_].

Like CsTi_8_F_33_ [46], [Xe_2_F_3_][Ti_8_F_33_] [45] also exhibits a layered structure. However, the 2-D polymeric ([Ti_8_F_33_]^−^)_∞_ anion in [Xe_2_F_3_][Ti_8_F_33_] has a different geometry (Figure 59) than in CsTi_8_F_33_. A basic structural motif resembles an oligomeric cubic [Ti_8_F_36_]^4−^ anion (Figure 20), which consists of eight TiF_6_ octahedra. These octameric units are connected by six common fluoride vertices and form a layered anion. The [Xe_2_F_3_]^+^ cations are located in a semi-closed channel. CsTi_8_F_33_ and [Xe_2_F_3_][Ti_8_F_33_] are the other examples containing the anion, which can be expressed by the same general formula [Ti_8_F_33_]^−^, but have a different geometry.

**Figure 60:**
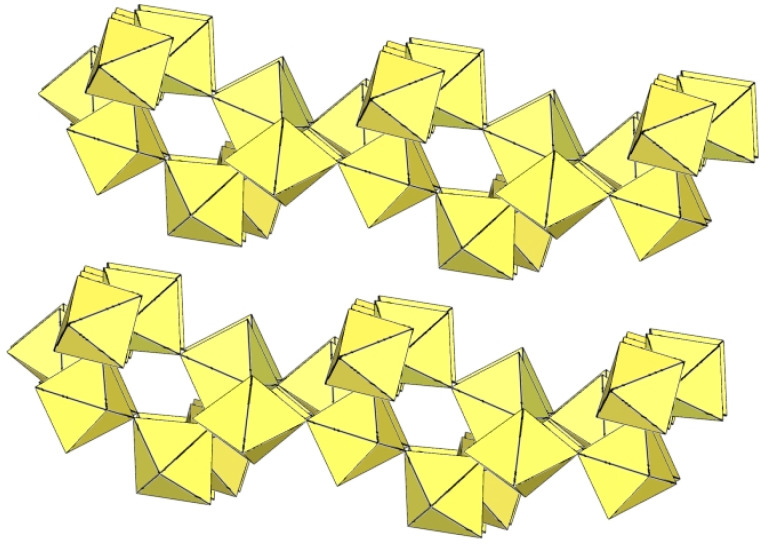
Packing of polymeric anionic layers ([Cr_2_F_9_]^−^)_∞_ in the crystal structure of XeF_2_·2CrF_4_.

Similar to XeF_2_·CrF_4_ [28], the determined Xe–F bond lengths in XeF_2_·2CrF_4_ indicate that XeF_2_ is at the beginning of its ionization pathway (XeF_2_ → [XeF]^+^+F^−^). Therefore, the formulation of the compound as the adduct XeF_2_·2CrF_4_ is more suitable than the ionic formulation [XeF^+^][Cr_2_F_9_] [37]. The basic structural unit is formed by four independent Cr atoms, each of which is octahedrally coordinated by six F atoms. To complete the octahedral coordination, two additional fluorine ligands are provided by two different XeF_2_ molecules. The distorted CrF_6_ octahedra are connected by common F atoms and form a layered structure (Figure 60).

## 7. Polymeric ([M_6_F_27_]^3−^)_∞_ Anion in the Form of Three-Dimensional Framework (M = Ti)

A summary of the crystal data of the salt consisting of polymeric ([M_6_F_27_]^3−^)_∞_ anion (M = Ti) in the form of a three-dimensional framework is given in Table 17.

Slow decomposition in attempts to grow single crystals of K_4_[Ti_8_F_36_]·8HF and Rb_4_[Ti_8_F_36_]·6HF [24] led to the growth of cube-shaped crystals of ([Ti_6_F_27_]^3−^)_∞_ salts. Later, the same type of anion ([Ti_6_F_27_]^3−^)_∞_ was found in [H_3_O]_3_[Ti_6_F_27_] [31]. Unfortunately, in all three cases, there is a problem with charge balance, i.e., a deficit of cations. For K and Rb salts, there is a possibility that some [H_3_O]^+^ was present, leading to mixed-cation A^+^/[H_3_O]^+^ salts.

**Figure 61:**
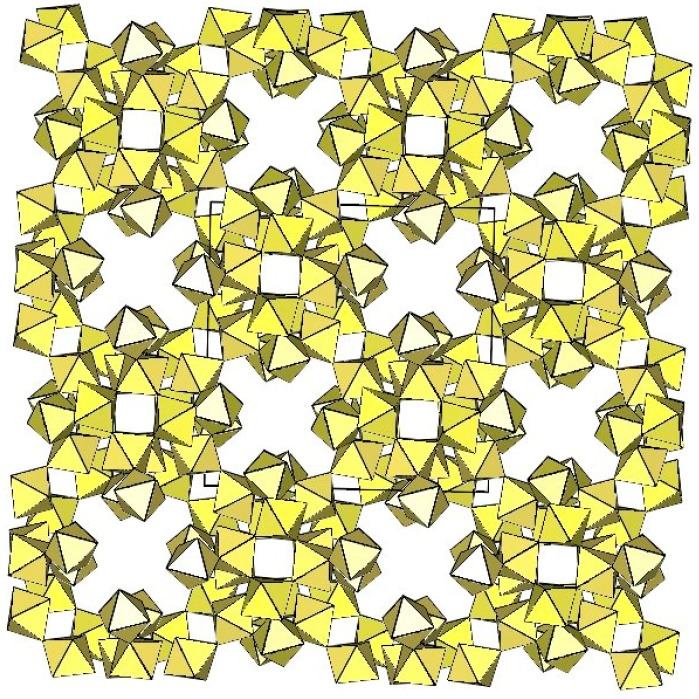
Three-dimensional framework of the ([Ti_6_F_27_]^3−^)_∞_ anion in the crystal structure of [H_3_O]_3_[Ti_6_F_27_].

The ([Ti_6_F_27_]^3−^)_∞_ anion is a three-dimensional framework consisting of TiF_6_ octahedra (Figure 61). Its structure can be described as composed of non-planar tetrameric Ti_4_F_20_ units consisting of four octahedra, each sharing two cis-vertices. Each Ti_4_F_20_ unit is connected to four other Ti_4_F_20_ units so that each TiF_6_ octahedron of a tetrameric ring is connected to another tetrameric unit. There are two types of channels in the crystal structure of the ([Ti_6_F_27_]^3−^)_∞_ anion. The channels are occupied by cations and probably also by molecules of the solvent.

## 8. Conclusions

On the basis of this review is possible to draw some conclusions and determine the further direction of this work:

Among the fluoridometallates (IV), the largest number of different anions is known for Ti. This is not so surprising in view of the numerous studies that have been carried out in recent years [5,6,7,11,12,14,15,16,19,22,23,24,26,32]. The use of some other asymmetrical organic cations could still lead to new anions with hitherto unknown geometry. The examples of Zr and Hf salts are limited to a single case for each element [16]. Since both elements prefer a higher coordination than six, it is not very likely that many new examples will be prepared.

[H_3_N(CH_2_)_2_NH_2_][VF_5_] is a unique example of a structurally characterized V(IV) fluoride compound that does not contain only an isolated [VF_6_]^2−^ anion [33]. Therefore, the chemistry of hybrid compounds with V(IV) is still an unexplored area. In fluorides, vanadium occurs in different oxidation states, ranging from +2 to +5. This could be an obstacle on the way to synthesizing inorganic or hybrid V(IV) fluorides. V(IV)could be reduced, oxidized, or disproportionated, resulting in V(III) and V(V) salts instead of the desired V(IV) salts. There are only a few examples of Nb(IV) fluorides (all are [NbF_6_]^2−^ salts [47,48]), while TaF_4_ and Ta(IV) fluorides are not known at all. Therefore, these two elements are not good candidates for the preparation of new Nb(IV) and Ta(IV) fluoride polyanions.

The chemistry of Cr(IV) polyanions is limited to salts with inorganic cations such as alkali metals and noble gas fluoride cations [15,28,37]. Due to the oxidizing power of Cr(IV), it is not very likely that many new hybrid polyfluoridechromates (IV) could be prepared. It is interesting to note that the [W_4_F_18_]^2−^ salt [WCl_2_(cp)_2_][W_4_F_18_] (cp = η-C_6_H_5_)] is an example of a W(IV) fluoride salt [20], while [WF_6_]^2−^ salts are not known. The Mo(IV) fluoride salts are rare and are limited to [MoF_6_]^2−^ salts [49]. Therefore, these three elements are also not very promising candidates for the preparation of new M(IV) fluoride polyanions (M = Cr, Mo, W).

For similar reasons as for Cr(IV), Mn(IV) is not a good choice for the preparation of new M(IV) (M = Mn) fluoride polyanions.

The M(IV) fluorides (M = Re, Ru, Os, Rh, Ir, Pd, Pt) are limited to [MF_6_]^2−^ salts, and no association of MF_6_ octahedra has been observed so far.

In the case of M(IV) (M = Si, Ge, Sn, Pb), there are a number of reports in which selected anions have been observed in solution or suggested by vibrational spectroscopy in the solid state, but the determination of their crystal structures in the solid state is still pending:

(1) Multinuclear NMR spectroscopy (^19^F, ^119^Sn) of N_5_SnF_5_ in aHF solution showed that the [SnF_5_]^−^ anion exists as both a dimeric oligomer [Sn_2_F_10_]^2−^ and an oligomeric cyclic tetramer [Sn_4_F_20_]^4−^ [39].
(2) The vibrational spectra of solid NF_4_SnF_5_ and NF_4_GeF_5_ are very similar to those of tetrameric NbF_5_ and TaF_5_, indicating the possible presence of [M_4_F_20_]^2−^ tetramers in NF_4_SnF_5_ and NF_4_GeF_5_ [40].
(3) The nature of N_2_FSn_2_F_9_ is still open. The anion [Sn_2_F_9_]^−^ most likely does not have a monomeric structure, but is probably present as an oligomer or polymer [41].
(4) The geometries of the anions in the salts N_2_F_3_SnF_5_, NF_4_Ti_2_F_9_, NF_4_Ti_3_F_13_, and NF_4_Ti_6_F_25_ are unknown [42,50].

Therefore, these elements (especially Sn and Pb) are the most promising for the synthesis of hybrid salts with new fluoridometallate (IV) polyanions.

Although examples of [MF_6_]^2^ salts are known for M = Ce [51], U [51], and Tc [51], it is not very likely that new fluoride polyanions will be synthesized in their case.

We can therefore assume that various oligomeric and polymeric anions still need to be prepared and structurally characterized.

## Figures and Tables

**Table 1:** Crystal data of the salts consisting of [M_2_F_11_]^3−^ anions (M = Ti, Cr).

Compound	Space Group	*a*, *b*, *c*/Å	*α*, *β*, *γ*/°	*V*/Å^3^	*Z*	*T*/K *	Ref.
[C_3_H_5_N_2_]_3_[Ti_2_F_11_]	monoclinic	13.5371(2)	90	3563.0(1)	8	200	[12]
	*C*2/m	25.7451(4)	100.980(1)				
		10.4139(2)	90				
[C_5_H_6_N]_2_[H_3_O][Ti_2_F_11_]·H_2_O	triclinic	6.684(5)	84.733(5)	454.1	1	293	[13]
	*P*-1	8.215(5)	85.250(5)				
		8.345(5)	86.692(5)				
[N(CH_3_)_4_]_4_[Ti_2_F_11_][Ti_2_F_9_(H_2_O)_2_]	monoclinic	10.7241(4)	90	870.82(5)	2	150	[14]
	*C*2	13.7028(5)	90.169(4)				
		5.9260(2)	90				
K_3_Cr_2_F_11_·2HF	monoclinic	11.694(8)	90	1114.9(13)	4	200	[15]
	*P*2_1_/*n*	7.541(4)	111.102(14)				
		13.552(10)	90				

* The crystal structures were determined at the indicated temperatures.

**Table 2:** Crystal data of the salt consisting of [M_3_F_15_]^3−^ anions (M = Zr).

Compound	Space Group	*a*, *b*, *c*/Å	*α*, *β*, *γ*/°	*V*/Å^3^	*Z*	*T*/K *	Ref.
[C_27_H_37_N_2_]_3_[Zr_3_F_15]_·4thf · 0.55(CH_2_Cl_2_)	triclinic	15.935(4)	81.698(16)	5401(3)	2	120	[16]
	*P*-1	17.240(4)	85.337(17)				
		21.679(7)	66.454(13)				

* The crystal structure was determined at the indicated temperature.

**Table 3:** Crystal data of the salts consisting of [M_3_F_16_]^4−^ anions (M = Ge).

Compound	Space Group	*a*, *b*, *c*/Å	*α*, *β*, *γ* /°	*V*/Å^3^	*Z*	*T*/K *	Ref.
[(CH_2_)_2_SOH][Ge_3_F_16_]	monoclinic	7.9406(11)	90	3094	2	123(2)	[17]
	*P*2_1_/*c*	27.224(2)	90.00				
		7.8817(11)	90				
[C(NH_2_)_2_(NH_3_)_2_][Ge_3_F_16_]·HF	tetragonal	12.000(5)	90	1617.1(12)	4	143(2)	[18]
	*P*4_2_*bc*	12.000(5)	90				
		11.230(5)	90				
[C(NH_2_)_2_(NH_3_)_2_][Ge_3_F_16_]·2HF	triclinic	7.3073(5)	86.360(7)	429.46(6)	1	143(2)	[18]
	*P*-1	7.4883(6)	80.768(6)				
		8.2439(7)	74.743(6)				

* The crystal structures were determined at the indicated temperatures.

**Table 4:** Crystal data of the salts consisting of [M_4_F_18_]^2−^ anions (M = Ti, W).

Compound	Space Group	*a*, *b*, *c*/Å	*α*, *β*, *γ* /°	*V*/Å^3^	*Z*	*T*/K *	Ref.
[TiF_2_([15]crown-5)][Ti_4_F_18_]⋅0.5MeCN	monoclinic	8.3335(9)	90	5560.4(11)	8	198	[5]
	*P*2_1_/*c*	41.887(5)	103.927(2)				
		16.412(2)	90				
[N(CH_3_)_4_]_2_[Ti_4_F_18_]	orthorhombic	13.278(1)	90	2431.1(3)	4	200	[6]
	*Pnma*	10.4935(6)	90				
		17.448(1)	90				
[(C_6_H_5_)_4_P]_2_[Ti_4_F_18_]	triclinic	10.1172(1)	83.880(9)	2544.4(2)	2	200	[6]
	*P*-1	13.0011(3)	80.335(8)				
		20.913(1)	69.988(6)				
[o-C_6_H_4_(P(C_6_H_5_)_2_H)_2_][Ti_4_F_18_]	monoclinic	15.264(2)	90	3698.8(6)	4	120	[19]
	*P*2_1_/*n*	14.925(2)	104.312(7)				
		16.747(2)	90				
[WCl_2_(cp)_2_][W_4_F_18_] (cp = η-C_6_H_5_)	orthorhombic	13.625(5)	90	3418(2)	8	296(1)	[20]
	*Pnma*	11.225(3)	90				
		22.350(3)	90				

* The crystal structures were determined at the indicated temperatures.

**Table 5:** Crystal-data of the salt consisting of [M_4_F_19_]^3−^ anions (M = Ti).

Compound	Space Group	*a*, *b*, *c*/Å	*α*, *β*, *γ*/°	*V*/Å^3^	*Z*	*T*/K *	Ref.
[XeF_5_]_3_[Ti_4_F_19_]	monoclinic	12.0866(5)	90	2416.6(2)	4	200	[21]
	*P*2_1_/*c*	9.5615(3)	96.301(2)				
		21.0377(8)	90				

* The crystal structure was determined at the indicated temperature.

**Table 6:** Crystal data of the salts consisting of [M_4_F_20_]^4−^ anions (M = Ti).

Compound	Space Group	*a*, *b*, *c*/Å	*α*, *β*, *γ*/°	*V*/Å^3^	*Z*	*T*/K *	Ref.
*α*-[C_3_H_5_N_2_]_4_[Ti_4_F_20_]	triclinic	8.791(3)	118.808(8)	681.8(5)	1	200	[12]
	*P*-1	9.971(4)	92.366(3)				
		10.126(4)	113.595(8)				
*β*-[C_3_H_5_N_2_]_4_[Ti_4_F_20_]	monoclinic	13.2139(4)	90	1384.35(10)	2	298	[12]
	*C2/m*	15.2096(7)	129.690(1)				
		8.9514(3)	90				
[C(NH_2_)_3_]_4_[Ti_4_F_20_]	triclinic	8.6958(2)	118.467(3)	636.42(3)	1	200	[22]
	*P*-1	9.7433(2)	111.687(3)				
		9.7533(3)	95.516(2)				
[C(NH_2_)_3_]_4_(H_3_O)_4_[Ti_4_F_20_][TiF_5_]_4_	monoclinic	9.5935(4)	90	2171.0(2)	2	150	[22]
	*P*2_1_/*c*	7.4536(4)	90.244(4)				
		30.361(1)	90				

* The crystal structures were determined at the indicated temperatures.

**Table 7:** Crystal data of the salt consisting of [M_5_F_23_]^3−^ anions (M = Ti).

Compound	Space Group	*a*, *b*, *c*/Å	*α*, *β*, *γ* /°	*V*/Å^3^	*Z*	*T*/K *	Ref.
[C_3_H_5_N_2_]_3_[Ti_5_F_23_]	orthorhombic	22.0259(4)	90	2784.29(9)	4	200	[12]
	*Pna*2_1_	10.2622(2)	90				
		12.3180(2)	90				

* Crystal structure was determined at the given temperature.

**Table 8:** Crystal data of the salts consisting of [M_6_F_27_]^3−^ anions (M = Ti).

Compound	Space Group	*a*, *b*, *c*/Å	*α*, *β*, *γ* /°	*V*/Å^3^	*Z*	*T*/K *	Ref.
C(NH_2_)_3_]_3_[Ti_6_F_27_]·SO_2_	monoclinic	18.0595(3)		6240.5(2)	8	150	[22]
	*P*2_1_/*c*	12.6281(2)	99.744(2)				
		27.7642(5)					
[C_3_H_5_N_2_]_2_[H_3_O][Ti_6_F_27_]	tetragonal	22.1506(4)	90	5686.1(2)	8	150	[22]
	*P*4_2_/*nmc*	22.1506(4)	90				
		11.5890(3)	90				

* The crystal structures were determined at the indicated temperatures.

**Table 9:** Crystal data of the salts consisting of [M_8_F_36_]^4−^ anions (M = Ti, Mn).

Compound	Space Group	*a*, *b*, *c*/Å	*α*, *β*, *γ*/°	*V*/Å^3^	*Z*	*T*/K *	Ref.
K_4_Ti_8_F_36_·8HF	triclinic	10.2054(7)	79.808(14)	886.21(14)	1	200	[24]
	*P*-1	10.3448(1)	65.208(11)				
		10.5896(2)	60.889(11)				
Rb_4_Ti_8_F_36_·6HF	triclinic	10.199(2)	89.68(6)	908.2(7)	1	200	[24]
	*P*-1	10.4191(5)	66.41(5)				
		10.5848(7)	64.17(4)				
[H_5_O_2_]_4_[Ti_8_F_36_]	tetragonal	11.3935(5)	90	1613.7(2)	2	150	[22]
	*I*4/*m*	11.3935(5)	90				
		12.4312(9)	90				
[XeF_5_]_4_[Mn_8_F_36_]	monoclinic	9.34476(12)	90	1974.98(4)	2	150	[25]
	*P*2_1_/*c*	17.9511(2)	99.5339(12)				
		11.93831(15)	90				

* The crystal structures were determined at the indicated temperatures.

**Table 10:** Crystal data of the salt consisting of [M_10_F_45_]^5−^ anions (M = Ti).

Compound	Space Group	*a*, *b*, *c*/Å	*α*, *β*, *γ*/°	*V*/Å^3^	*Z*	*T*/K *	Ref.
*α*-[XeF_5_]_5_[Ti_10_F_45_]	monoclinic	18.9017(6)	90	5436.4(3)	4	150	[26]
	*Cc*	16.6334(5)	94.004(3)				
		17.3336(5)	90				
*β*-[XeF_5_]_5_[Ti_10_F_45_]	orthorhombic	18.8980(4)	90	5489.7(2)	4	296	[26]
	*Cmc2_1_*	16.7388(4)	90				
		17.3542(4)	90				

* The crystal structure was determined at the indicated temperature.

**Table 11:** Crystal data of the salts consisting of trans-([MF_5_]^−^)_∞_ anions (M = Ge, Cr).

Compound	Space Group	*a*, *b*, *c*/Å	*α*, *β*, *γ*/°	*V*/Å^3^	*Z*	*T*/K *	Ref.
XeF_5_GeF_5_	orthorhombic	7.119(2)	90	683.9(5)	4	293	[27]
	*Pmnb*	12.986(4)	90				
		7.398(1)	90				
XeF_2_·CrF_4_	monoclinic	7.666(2)	90	551.5	4	293(1)	[28]
	*P*2_1_/*n*	7.268(5)	91.25(2)				
		9.901(3)	90				

* The crystal structures were determined at the indicated temperatures.

**Table 12:** Crystal data of the salts consisting of *cis*-([MF_5_]^−^)_∞_ anions (M = Ti, V, Cr, Mn, Ge, Sn, Pb).

Compound	Space Group	*a*, *b*, *c*/Å	*α*, *β*, *γ* /°	*V*/Å^3^	*Z*	*T*/K *	Ref.
H_3_OTiF_5_	monoclinic	14.528(5)	90	874.9	8	RT **	[29]
	*C*2/*c*	4.839(l)	115.59(5)				
		13.798(5)	90				
NH_4_TiF_5_	monoclinic	14.683(1)	90	1829.9(3)	4	293(2)	[30]
	*P2_1_/n*	6.392(l)	110.538(2)				
		20.82(2)	90				
NaTiF_5_·HF	monoclinic	15.1768(9)	90	1004.2(1)	8	200	[31]
	*C*/2*c*	6.4171(3)	108.266(2)				
		10.8580(7)	90				
KTiF_5_	monoclinic	20.277(3)	90	1681.9(4)	16	157	[31]
	*C*/2*c*	6.1768(8)	110.960(9)				
		14.380(2)	90				
KTiF_5_·HF	monoclinic	13.671(2)	90	1020.9(2)	8	200	[31]
	*C*/2*c*	8.1382(6)	114.217(4)				
		10.061(1)	90				
RbTiF_5_·HF	monoclinic	13.823(6)	90	1072.1(8)	8	150	[31]
	*C*/2*c*	8.295(3)	114.35(2)				
		10.264(5)	90				
CsTiF_5_	orthorhombic	5.3986(2)	90	487.97(3)	4	150	[31]
	*Pnam*	14.0057(5)	90				
		6.4536(3)	90				
[C_2_H_4_(NH_3_)_2_](TiF_5_)_2_	monoclinic	5.7801(3)	90	489.06(5)	2	200	[32]
	*P*2_1_/*c*	15.447(1)	92.433(5)				
		5.4825(3)	90				
[H_3_N(CH_2_)_2_NH_2_][VF_5_]	orthorhombic	10.5231(9)	90	609.70(9)	4	90(2)	[33]
	*Pnma*	5.7185(5)	90				
		10.1319(8)	90				
KCrF_5_	orthorhombic	5.425(2)	90	395.8(2)	-	200	[15]
	-	7.427(2)	90				
		9.824(4)	90				
RbCrF_5_	orthorhombic	5.5150(17)	90	429.7(8)	4	200	[15]
	*Pmc*2_1_	7.653(14)	90				
		10.181(5)	90				
CsCrF_5_	orthorhombic	10.70(2)	90	476.5(14)	4	200	[15]
	*Pnma*	5.611(8)	90				
		7.936(11)	90				
O_2_GeF_5_·HF	monoclinic	9.8444(8)	90				[34]
	*I*2/*a*	8.0274(6)	110.774(10)	968.14(15)	8	150	
		13.1030(12)	90				
ClO_2_SnF_5_	monoclinic	7.3673(4)	90	507.354	4	100	[35]
	*P*2_1_/*n*	5.1042(3)	93.026(2)				
		13.5108(8)	90				
ClOF_2_SnF_5_	monoclinic	15.828(3)	90	555.7(2)	4	100	[36]
	*C*2	5.0614(10)	111.25(3)				
		7.4425(15)	90				
ClOF_2_PbF_5_	monoclinic	16.1838(12)	90	583.29(7)	4	100	[36]
	*C*2	5.1546(4)	111.932(2)				
		7.5376(5)	90				
XeF_5_TiF_5_	orthorhombic	18.139(2)	90	2810.0(5)	16	150	[26]
	*Pbca*	8.5173(9)	90				
		18.1876(16)	90				
XeF_5_CrF_5_	orthorhombic	18.281(13)	90	2854(4)	16	268(2)	[37]
	*Pbca*	8.429(7)	90				
		18.521(12)	90				
XeF_5_MnF_5_	monoclinic	9.0265(5)	90	1348.4(2)	2	120	[25]
	*P*2_1_/*c*	17.8898(9)	90.132(5)				
		8.3506(5)	90				
ClO_2_GeF_5_	orthorhombic	14.6480(15)	90	987.0(4)	8	168(10)	[27]
	*C*222_1_	7.5762(11)	90				
		8.8941(15)	90				

* The crystal structures were determined at the indicated temperatures.** Measured at room temperature. The exact temperature was not reported.

**Table 13:** Crystal data of the salt consisting of *cis*- and *trans*-([MF_5_]^−^)_n_ anions (M = Cr).

Compound	Space Group	*a*, *b*, *c*/Å	*α*, *β*, *γ*/°	*V*/Å^3^	*Z*	*T*/K *	Ref.
(XeF_5_CrF_5_)_4_·XeF_4_	orthorhombic	11.988(6)	90	3144.8	4	293(1)	[28]
	*Pbca*	15.862(2)	90				
		16.538(2)	90				

* The crystal structure was determined at the indicated temperature.

**Table 14:** Crystal data of the salts consisting of double chain-like ([M_2_F_9_]^−^)_∞_ anions (M = Ti, Mn, Sn).

Compound	Space Group	*a*, *b*, *c*/Å	*α*, *β*, *γ*/°	*V*/Å^3^	*Z*	*T*/K *	Ref.
*α*-O_2_Sn_2_F_9_	orthorhombic	4.0473(3)	90	371.63(4)	2	200	[34]
	*Immm*	8.0199(4)	90				
		11.4491(8)	90				
*α*-[H_3_O][Ti_2_F_9_]	orthorhombic	8.988(4)	90	722.6(5)	4	100	[6]
	*Pnma*	5.451(2)	90				
		14.748(6)	90				
*β*-[H_3_O][Ti_2_F_9_]	monoclinic	5.3178(2)	90				[22]
	*P*2_1_/c	16.0786(8)	91.440(3)	756.10(6)	4	150	
		8.8459(3)	90				
NaTi_2_F_9_·HF	orthorhombic	5.3084(3)	90	740.98(7)	4	200	[31]
	*Pnma*	10.0736(6)	90				
		13.8566(8)	90				
RbTi_2_F_9_	monoclinic	15.0380(7)	90	1480.5(1)	8	157	[31]
	*P*2_1_/*c*	5.3244(3)	93.788(5)				
		18.531(1)	90				
CsTi_2_F_9_	monoclinic	1136.3(3)	90	798.2(4)	4	200	[6]
	*C*2/*c*	1471.1(3)	116.41(2)				
		533.18(14)	90				
*α-*[C_3_H_5_N_2_][Ti_2_F_9_]	monoclinic	5.3914(3)	90	997.78(11)	4	200	[12]
	*P*2_1_/*a*	15.4836(10)	90.977(4)				
		11.9543(8)	90				
*β-*[C_3_H_5_N_2_][Ti_2_F_9_]	orthorhombic	5.3978(2)	90	1004.63(8)	4	298	[12]
	*Pnma*	12.2169(6)	90				
		15.2345(7)	90				
[C(NH_2_)_3_][Ti_2_F_9_]	orthorhombic	5.4001(2)	90	952.87(9)	4	200	[22]
	*Pnma*	11.9123(5)	90				
		14.813(1)	90				
[ClO_2_][Ti_2_F_9_]	monoclinic	11.084(2)	90	801.4(2)	4	100	[35]
	*C*2/*c*	14.603(2)	111.73(1)				
		5.330(1)	90				
O_2_Mn_2_F_9_	monoclinic	17.55	90	1306.8	8	123	[43]
	*C*2/*c*	8.37	102.3				
		9.10	90				

* The crystal structures were determined at the indicated temperatures. The exact temperature was not reported.

**Table 15:** Crystal data of the salts consisting of polymeric column-like ([M_3_F_13_]^−^)_∞_, ([M_4_F_19_]^3−^)_∞_, ([M_7_F_30_]^2−^)_∞_, and ([M_9_F_38_]^2−^)_n_ anions (M = Ti).

Compound	Space Group	*a*, *b*, *c*/Å	*α*, *β*, *γ* /°	*V*/Å^3^	*Z*	*T*/K *	Ref.
[XeF_5_][Ti_3_F_13_]	triclinic	9.7699(6)	89.601(5)	1327.82(14)	4	150	[26]
	*P-1*	11.0276(6)	69.992(5)				
		13.4581(7)	77.717(5)				
Cs_3_[Ti_4_F_19_]	orthorhombic	5.3999(4)	90	1771.7(2)	4	150	[31]
	*Cmcm*	15.2661(12)	90				
		21.4921(15)	90				
(O_2_)_2_[Ti_7_F_30_]	trigonal	10.19(2)	90	584.7	1	153	[44]
	*P*-3	10.19(2)	90				
		6.50(0)	120				
[XeF]_2_[Ti_9_F_38_]	monoclinic	17.5967(8)	90	3072.9(2)	4	150	[45]
	*Cc*	15.3862(6)	108.2795(16)				
		11.9529(6)	90				

* The crystal structures were determined at the indicated temperatures.

**Table 16:** Crystal data of the salts consisting of layered ([M_8_F_33_]^−^)_∞_ and ([M_2_F_9_]^−^)_∞_ anions (M = Ti, Cr).

Compound	Space Group	*a*, *b*, *c*/Å	*α*, *β*, *γ* /°	*V*/Å^3^	*Z*	*T*/K *	Ref.
CsTi_8_F_33_	trigonal	8.622(5)	90	1224.5	2	RT **	[46]
	*P31c*	8.622(5)	90				
		19.02(1)	120				
[Xe_2_F_3_][Ti_8_F_33_]	monoclinic	17.6347(5)	90	2929.1(1)	4	150	[45]
	*P2/a*	8.4106(2)	97.140(1)				
		19.9028(5)	90				
XeF_2_·2CrF_4_	triclinic	8.551(3)	76.02(2)	789.7(4)	4	293(2)	[37]
	*P*-1	9.221(3)	81.36(2)				
		10.438(3)	88.08(3)				

* Crystal structures were determined at the indicated temperatures. ** Measured at room temperature. The exact temperature was not reported.

**Table 17:** Crystal data of the salt consisting of polymeric ([M_6_F_27_]^3−^)_∞_ anion (M = Ti) in the form of a three-dimensional framework.

Compound	Space Group	*a*, *b*, *c*/Å	*α*, *β*, *γ*/°	*V*/Å^3^	*Z*	*T*/K *	Ref.
[H_3_O]_3_[Ti_6_F_27_]	cubic	17.2014(9)	90	5089.7(8)	8	150	[31]
	*Pn-3n*	17.2014(9)	90				
		17.2014(9)	90				

* The crystal structure was determined at the indicated temperature.
